# PML::RARA and GATA2 proteins interact via DNA templates to induce aberrant self-renewal in mouse and human hematopoietic cells

**DOI:** 10.1073/pnas.2317690121

**Published:** 2024-04-22

**Authors:** Casey D. S. Katerndahl, Olivia R. S. Rogers, Ryan B. Day, Ziheng Xu, Nichole M. Helton, Sai Mukund Ramakrishnan, Christopher A. Miller, Timothy J. Ley

**Affiliations:** ^a^Division of Oncology, Department of Internal Medicine, Section of Stem Cell Biology, Washington University School of Medicine, St. Louis, MO 63110

**Keywords:** acute myeloid leukemia, acute promyelocytic leukemia, PML::RARA, GATA2, self-renewal

## Abstract

The underlying mechanism(s) by which the PML::RARA fusion protein initiates acute promyelocytic leukemia is not yet clear. We defined the genomic binding sites of PML::RARA in primary mouse and human hematopoietic progenitor cells with V5-tagged PML::RARA, using anti-V5-PML::RARA chromatin immunoprecipitation sequencing and CUT&RUN approaches. Most genomic PML::RARA binding sites were found in regions that were already chromatin-accessible (defined by ATAC-seq) in unmanipulated, wild-type promyelocytes, suggesting that these regions are “open” prior to PML::RARA expression. We found that GATA binding motifs, and the direct binding of the chromatin “pioneering factor” GATA2, were significantly enriched near PML::RARA binding sites. Proximity labeling studies revealed that PML::RARA interacts with ~250 proteins in primary mouse hematopoietic cells; GATA2 and 33 others require PML::RARA binding to DNA for the interaction to occur, suggesting that binding to their cognate DNA target motifs may stabilize their interactions. In the absence of *PML::RARA*, *Gata2* overexpression induces many of the same epigenetic and transcriptional changes as *PML::RARA*. These findings suggested that *PML::RARA* may indirectly initiate its transcriptional program by activating *Gata2* expression: Indeed, we demonstrated that inactivation of *Gata2* prior to *PML::RARA* expression prevented its ability to induce self-renewal. These data suggested that GATA2 binding creates accessible chromatin regions enriched for both GATA and Retinoic Acid Receptor Element motifs, where GATA2 and PML::RARA can potentially bind and interact with each other. In turn, PML::RARA binding to DNA promotes a feed-forward transcriptional program by positively regulating *Gata2* expression. *Gata2* may therefore be required for *PML::RARA* to establish its transcriptional program.

## Significance

Several acute myeloid leukemia (AML) initiating mutations involve transcription factors and are thought to initiate the disease via epigenetic reprogramming. A better understanding of the mechanisms used by one such mutation, *PML::RARA*, may provide important insights for other AML-initiating events. Reprogramming of hematopoietic stem and progenitor cells by *PML::RARA* leads to aberrant self-renewal, a precursor to the development of APL (acute promyelocytic leukemia). We defined the DNA binding sites of PML::RARA in the genomes of primary hematopoietic progenitors, which revealed that *PML::RARA* reprograms these cells in cooperation with the transcription factor *Gata2*; further, *Gata2* is required for *PML::RARA* to epigenetically reprogram hematopoietic progenitors and initiate aberrant self-renewal.

Acute promyelocytic leukemia (APL) is initiated by the *PML::RARA* fusion gene in greater than 95% of cases (1, 2). All-trans retinoic acid (ATRA) and arsenic trioxide combination therapy degrades the initiating protein (3–5) and leads to durable responses in more than 95% of favorable risk APL cases (6). However, the molecular mechanisms by which *PML::RARA* causes aberrant self-renewal and transformation are still poorly understood. Transgenic mice that express *PML::RARA* in early myeloid cells develop APL with long latency (7–11), suggesting that *PML::RARA* requires secondary mutations for progression; indeed, several cooperating mutations have been identified in APL (e.g., *FLT3, SPI1, GATA2,* WT*1, KDM6A,* and *RAS*, among others) (12–17). A model of how these mutations cooperate with *PML::RARA* and *GATA2* to cause APL (and a summary of this study) is shown in *SI Appendix*, Fig. S1.

PML::RARA is thought to act as a transcription factor that binds to repeats of RARE (Retinoic Acid Receptor Element) via the DNA binding domain of the transcription factor RARA (18, 19). Consistent with this role, we and others have shown that the ability of PML::RARA to block myeloid differentiation, and induce aberrant serial replating and transformation, depends on PML::RARA binding to DNA (12, 19, 20). However, there is no clear consensus on where PML::RARA binds in the genomes of early myeloid cells or APL cells. Three independent studies, using different technical approaches, reagents, and cellular substrates (*SI Appendix*, Fig. S2*A*) have previously been performed. A retrospective analysis of these studies (*SI Appendix*, Fig. S2*B*) reveals that only 89 of the thousands of binding sites identified are conserved among all three studies (21–23). Two of these studies relied on antibodies specific to PML and RARA, which could potentially identify the binding of endogenous PML and RARA to DNA, in addition to PML::RARA (21, 22). The third study used an antibody raised against a 200 amino acid region spanning the bcr1 fusion site of PML::RARA; 88 amino acids were derived from the PML portion of the fusion, and 112 were from RARA (23). Our evaluation of this antibody with western blotting did reveal an ability to recognize PML::RARA in APL cells with the bcr1 fusion, but it also recognizes nonspecific proteins of a similar molecular weight in primary human AML cells and human AML cell lines that do not contain the PML::RARA fusion (*SI Appendix*, Fig. S1*C*). Because of this ambiguity, we decided to use a more definitive approach to perform an independent analysis of the binding sites of PML::RARA in the genomes of both primary mouse and human hematopoietic progenitor cells, which were not examined in the previous studies. We coupled these analyses with studies of chromatin accessibility and gene expression in the same cells, to better define the downstream consequences of PML::RARA binding in the genome. Further, while PML::RARA has been shown to interact with a limited number of proteins using hypothesis-driven coimmunoprecipitation assays (24–30), the global protein interactions of PML::RARA in hematopoietic progenitors have not yet been systematically defined in an unbiased fashion. In this study, we established and integrated databases for all of these data layers and identified GATA2 as one important cofactor for PML::RARA-mediated transcriptional reprogramming of early myeloid progenitor cells.

## Results

### Identification of the Genomic Binding Sites of PML::RARA in Primary Hematopoietic Cells.

**Fig. 1.**
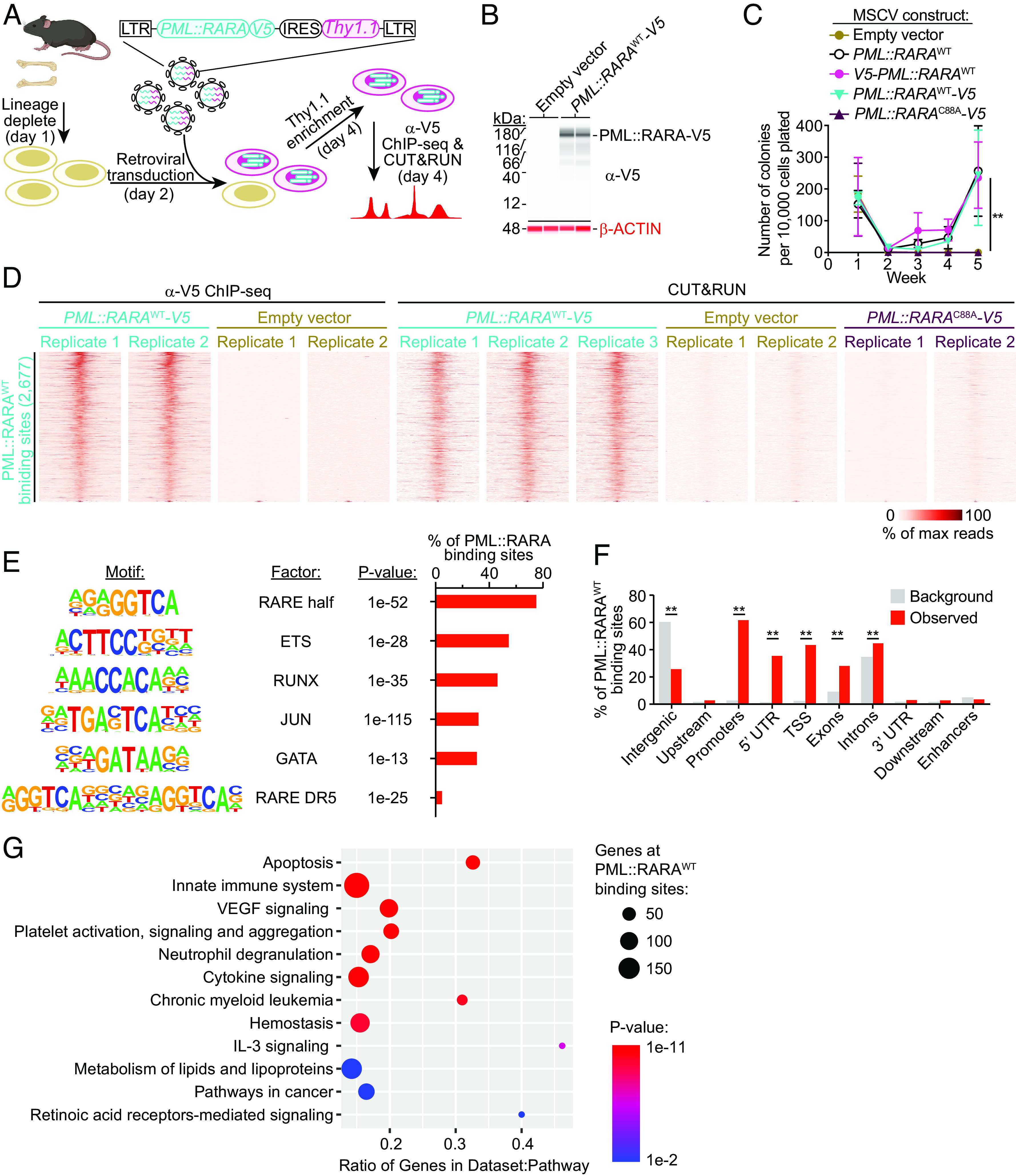
Identification of the binding sites of V5-tagged PML::RARA in primary hematopoietic cells by ChIP-seq and CUT&RUN. (*A*) Experimental schematic in which WT bone marrow cells were lineage-depleted and retrovirally transduced with MSCV vectors containing no insert (empty vector), WT *PML::RARA* cDNA (*PML::RARA*^WT^), *PML::RARA*^WT^ with a V5 epitope tag on the N terminus (*V5-PML::RARA*^WT^) or C terminus (*PML::RARA*^WT^*-V5*), or a V5-tagged C88A mutant in the RARA domain of *PML::RARA* (*PML::RARA*^C88A^*-V5*). Cells were grown in SCF, FLT3L, IL-3, and TPO for 2 d following transduction, and then, anti-V5 ChIP-seq and CUT&RUN were performed. (*B*) Anti-V5 (*Top* blot) or anti-beta ACTIN (*Bottom* blot) western blot analysis in cells transduced with *PML::RARA*^WT^*-V5* or an empty vector (*n* = 2, each), 2 d following transduction. Representative of 3 total replicates. (*C*) Colony counts from serial replating assays (*n* = 3, each). **P < 0.01 by two-way ANOVA between cells transduced with *PML::RARA*^WT^*, V5-PML::RARA*^WT^, or *PML::RARA*^WT^*-V5,* and those transduced with empty vector or *PML::RARA*^C88A^*-V5* at week 5. (*D*) “Tornado plots” of anti-V5 ChIP-seq (*Left* 4 panels) and CUT&RUN (*Right* 7 panels) at PML::RARA^WT^ binding sites plotted along the *Y*-axis. Each panel represents an independent biological replicate. Each row corresponds to one locus across all samples. (*E*) Motif enrichment at PML::RARA^WT^ binding sites by HOMER analysis (34). (*F*) Distribution of PML::RARA^WT^ binding sites at various regions in the genome compared to that of the mm10 reference genome (background). **P < 0.01. (*G*) Pathway enrichment at PML::RARA^WT^ binding sites. Genes within 1 kb of binding sites were analyzed.

To identify the genomic binding sites of PML::RARA in the chromatin of primary hematopoietic cells, we cloned a V5 epitope tag in-frame with PML::RARA. We then transduced lineage-depleted wild-type (WT) mouse bone marrow cells with retroviral murine stem cell virus (MSCV) vectors containing an internal ribosome entry site (IRES) followed by a Thy1.1 cDNA to allow for the purification of transduced cells. These vectors either contained no insert (“empty vector”), WT *PML::RARA* cDNA (*PML::RARA*^WT^), N- or C-terminally tagged *PML::RARA*^WT^ (*V5-PML::RARA*^WT^ and *PML::RARA*^WT^*-V5* respectively), or a V5-tagged C88A mutant in the *RARA* domain of *PML::RARA* (*PML::RARA*^C88A^*-V5*) (Fig. 1 *A* and *B* and *SI Appendix*, Table S1). C88 coordinates a Zn^2+^ ion within the *RARA* zinc-finger DNA binding domain, and its only described function is to facilitate binding to DNA (31, 32). The C88A mutation in *RARA* has previously been shown to abrogate the ability of RARA and PML::RARA to bind to DNA (19, 33). The V5 tag did not interfere with the ability of *PML::RARA*^WT^ to cause aberrant serial replating in methylcellulose colony forming assays; expression of *V5-PML::RARA*^WT^*, PML::RARA*^WT^*-V5,* and untagged *PML::RARA*^WT^ all led to similar colony counts after 5 wk of replating (Fig. 1*C*). In contrast, transduction with an empty vector, or *PML::RARA*^C88A^*-V5,* was unable to induce replating. This suggests that the ability of *PML::RARA* to induce aberrant self-renewal is dependent upon PML::RARA binding to DNA.

We then used this system to identify the genomic binding sites of PML::RARA^WT^ in lineage-depleted mouse bone marrow cells transduced with PML::RARA^WT^-V5, compared to empty vector transduced cells, using anti-V5 chromatin immunoprecipitation sequencing (ChIP-seq) 2 d following transduction; 5,481 binding sites were identified. To orthogonally validate these results, we used an anti-V5 CUT&RUN (35) approach, which identified 5,361 PML::RARA^WT^-V5 binding sites. By merging these datasets, 2,677 PML::RARA^WT^-V5 binding sites were identified by both techniques, representing orthogonally validated, high confidence regions for PML::RARA^WT^ binding (henceforth termed “PML::RARA^WT^ binding sites”) (Fig. 1*D* and *SI Appendix*, Fig. S3*A*). In contrast, PML::RARA^C88A^ bound to 22 sites by CUT&RUN, none of which were among the 2,677 PML::RARA^WT^ binding sites. This serves as an important negative control for true PML::RARA-mediated DNA binding events. Consistent with previous reports (18), the PML::RARA^WT^ binding sites were enriched for direct repeats of RARE with a 5 bp spacer (DR5) (4.7% of PML::RARA^WT^ binding sites) (Fig. 1*E*). However, PML::RARA^WT^ binding sites were much more enriched for regions containing RARE half sites (75.0% of PML::RARA^WT^ binding sites; Fig. 1*E*), suggesting that PML::RARA can also bind to RARE half motifs. PML::RARA^WT^ binding was highly enriched at promoters and transcriptional start sites (TSSs) (61.6, and 42.8% of sites, respectively; Fig. 1*F*) and was found within 1 kb of 1,850 genes (Dataset S1). These were enriched for genes involved in apoptosis, cytokine signaling, innate immunity, cancer, and retinoic acid receptor signaling (Fig. 1*G*). In addition, the genes bound by PML::RARA^WT^ included several that are known to be transcriptionally regulated by *PML::RARA* (12, 36–38), including *Gata2, Rarb, Spi1* (*Pu.1*), and *Cdkn2c*.

To validate these results, we expressed V5-PML::RARA using the same retroviral system in human CD34 enriched cord blood cells. Both V5-tagged and untagged PML::RARA were able to disrupt PML nuclear bodies into microspeckles (*SI Appendix*, Fig. S4 *A* and *B*). Anti-V5 ChIP-seq using this model identified 2,064 PML::RARA binding sites (*SI Appendix*, Fig. S5*A*). PML::RARA binding was completely eliminated upon treatment with ATRA for 48 h, which further suggests that the V5-PML::RARA ChIP-seq approach is highly specific. PML::RARA binding sites in human CD34 cells were similarly enriched for RARE half sites and DR5 motifs (72.0% and 5.23% of PML::RARA binding sites, respectively), occurred primarily at promoters or TSSs (66.8% and 27.5% of PML::RARA binding sites, respectively), and within 1 kb of 1,454 genes that were involved in similar pathways as those identified in mouse hematopoietic cells (*SI Appendix*, Fig. S5 *B*–*D* and Dataset S2). PML::RARA binding sites were found within 1 kb of 481 genes shared in mouse and human hematopoietic cells (*SI Appendix*, Fig. S5*E*). These data define the orthogonally verified, high-confidence genomic binding sites of PML::RARA shared in primary mouse and human hematopoietic cells.

### PML::RARA Overexpression Causes Changes in Gene Expression via Binding to DNA.

**Fig. 2.**
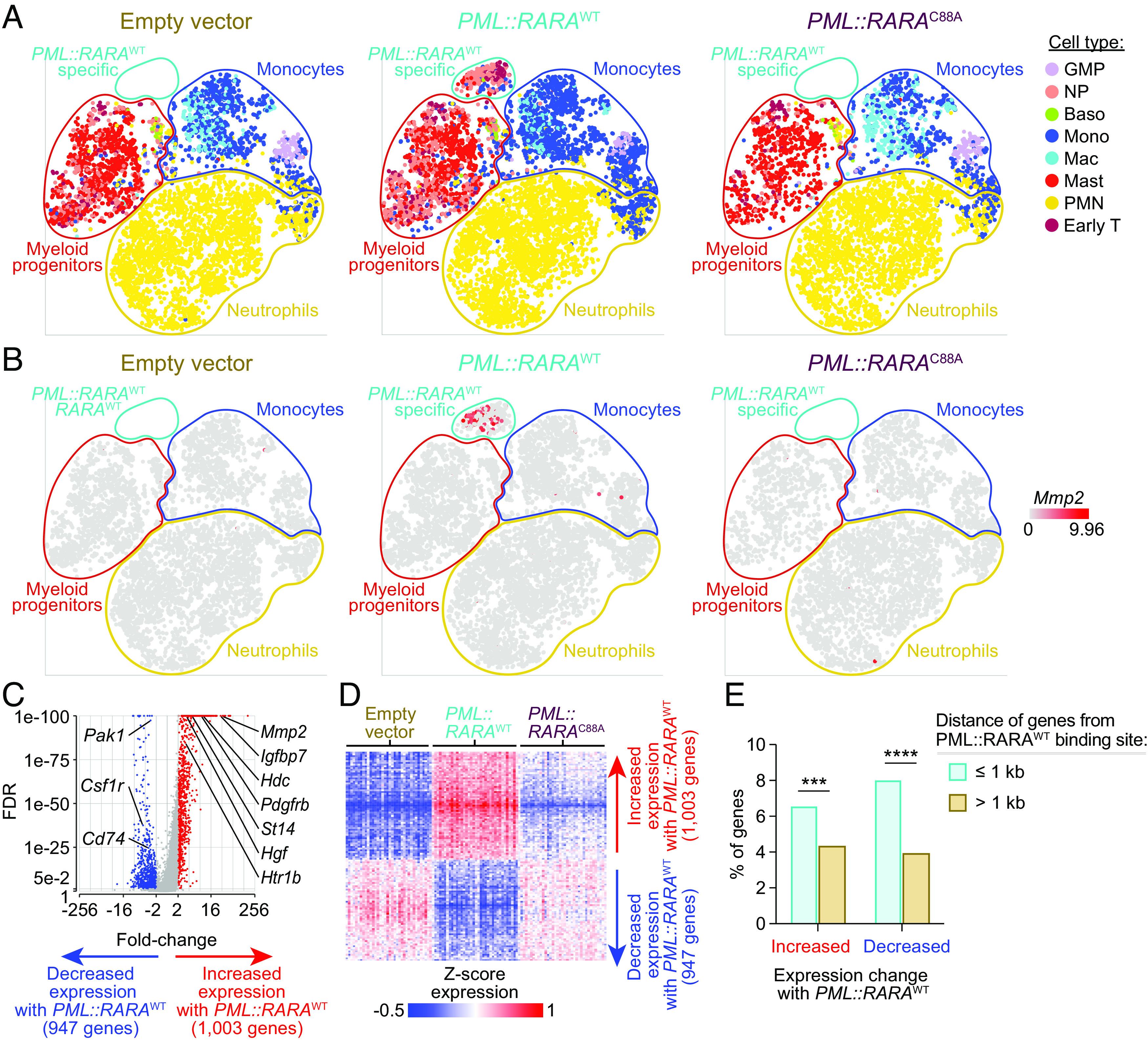
scRNA-seq following *PML::RARA* overexpression in mouse hematopoietic stem and progenitor cells. Lineage-depleted mouse bone marrow cells were transduced with MSCV-IRES-GFP-based retroviruses containing an empty vector, *PML::RARA*^WT^, or *PML::RARA*^C88A^. The cells were grown in SCF, FLT3L, IL-3, and TPO for an additional 7 d, at which point they were evaluated by scRNA-seq. (*A*) t-Distributed stochastic neighbor embedding (t-SNE) plots with lineage assignment based on Haemopedia gene expression profiling (41, 42). A unique population of myeloid precursor cells that are only present in cells transduced with *PML::RARA*^WT^ is outlined in light blue (“*PML::RARA*^WT^ specific”). NP = neutrophil progenitor. (*B*) t-SNE plots of the relative expression of *Mmp2* by scRNA-seq. (*C*) Volcano plot of expression changes between GFP+ *PML::RARA*^WT^ vs. empty vector transduced cells. (*D*) Heat map of the 1,995 DEGs in GFP+ *PML::RARA*^WT^ vs. empty vector transduced cells (FDR ≤ 0.05 and fold-change ≥ 2). *PML::RARA*^C88A^ transduced cells are passively plotted on the *Right*. (*E*) Bar graph of the percentage of genes within 1 kb or greater than 1 kb from a PML::RARA^WT^ binding site that show an increase or decrease in GFP+ *PML::RARA*^WT^ vs. empty vector transduced cells by scRNA-seq. **P < 0.01 and ***P < 0.001 by Fisher’s exact text.

To identify the short-term transcriptional consequences of *PML::RARA* expression, we performed single-cell RNA sequencing (scRNA-seq) on mouse bone marrow cells 7 d after transduction with MSCV-IRES-GFP (green fluorescent protein)-based retroviruses containing *PML::RARA*^WT^*, PML::RARA*^C88A^, or an empty vector. Consistent with our previous study (12), expression of *PML::RARA*^WT^ was associated with the development of a population of immature myeloid cells with a unique transcriptional profile that clustered separately from untransduced (GFP-) cells or cells transduced with empty vector or *PML::RARA*^C88A^ (Fig. 2 *A* and *B* and *SI Appendix*, Fig. S6 *A*–*E*). This analysis also identified 1,950 differentially expressed genes (DEGs) between *PML::RARA*^WT^ GFP+ cells and empty vector GFP+ cells (FDR ≤ 0.05 and fold-change ≥ 2) (Fig. 2 *C* and *D* and Dataset S3). 1,003 of these DEGs were up-regulated (including *Mmp2*) and 947 were down-regulated. 984 (50.5%) DEGs were dependent upon PML::RARA binding to DNA, since they were differentially expressed between *PML::RARA*^WT^ and *PML::RARA*^C88A^ transduced cells (Fig. 2*D* and Dataset S4). Further, 136 of these DNA binding–dependent *PML::RARA*^WT^ DEGs (78 up-regulated, 58 down-regulated) were found within 1 kb of a PML::RARA binding site: Such genes may be dysregulated due to direct PML::RARA binding nearby (Fig. 2*E*). To determine whether PML::RARA similarly regulates transcription in human hematopoietic cells, we performed scRNA-seq on human CD34+ cord blood cells 7 d after transduction with the same *PML::RARA* MSCV constructs. *PML::RARA*^WT^ expression also led to a population of cells with a unique transcriptional signature in this model (*SI Appendix*, Fig. S7 *A* and *B*). In addition, *PML::RARA*^WT^ expression was associated with 1,982 DEGs (1,424 up-regulated, including *GATA2*, and 558 down-regulated), of which 155 may represent direct targets of PML::RARA: These genes were dysregulated by *PML::RARA* in a DNA binding–dependent mechanism, and had a PML::RARA binding site within 1 kb; 135 of these 155 were up-regulated, 20 were down-regulated (FDR ≤ 0.05 and fold-change ≥ 2) (*SI Appendix*, Fig. S7 *C*–*E* and Datasets S5 and S6). Comparing the human and mouse datasets, 338 homologous genes were dysregulated by *PML::RARA* expression in both species (197 up-regulated, 141 down-regulated). One of these genes was *MMP2* (Fig. 2*B* and *SI Appendix*, Fig. S7*B*), which has previously been implicated in AML pathogenesis (39), and which also shows a 173-fold increase in expression in human APLs compared to healthy donor promyelocytes (*SI Appendix*, Fig. S7*F*) (40). Although the role of *MMP2* is unclear for APL pathogenesis, it is a good example of a gene that is specifically regulated by *PML::RARA* at a transcriptional and epigenetic level. Taken together, these data show that overexpression of *PML::RARA* in mouse or human hematopoietic cells leads to the altered expression of thousands of genes and that most of these changes are dependent upon PML::RARA binding to DNA.

### PML::RARA Expression Leads to Coordinate Changes in DNA Accessibility and Transcription.

**Fig. 3.**
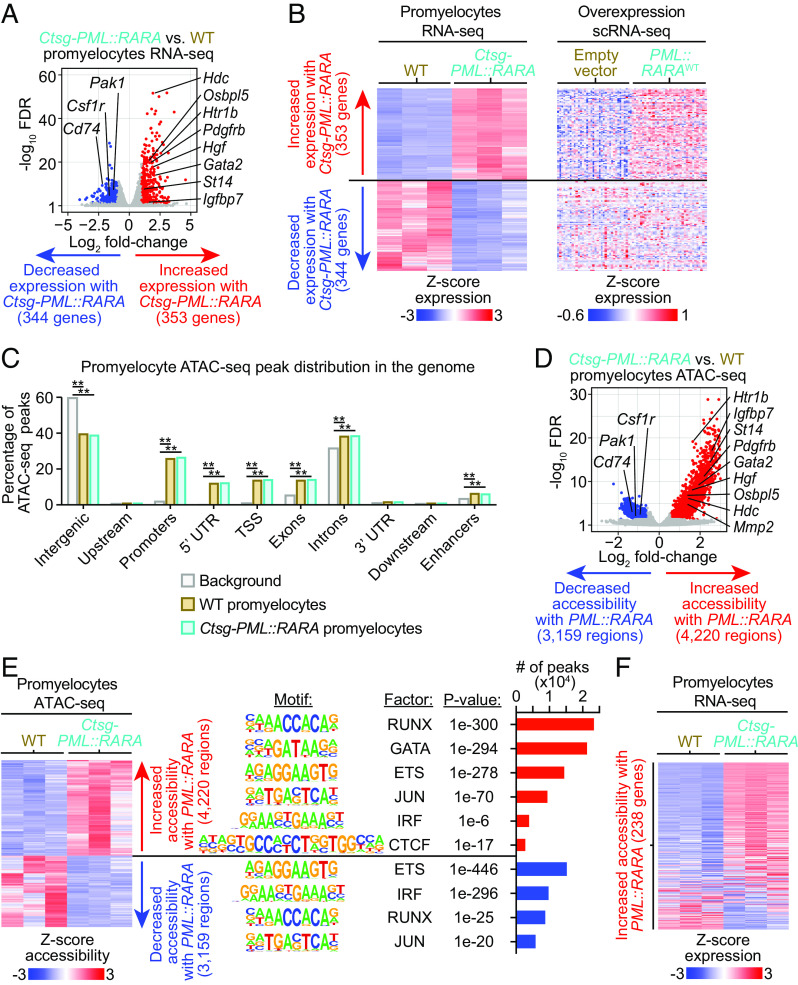
Transcriptional and epigenetic consequences of *PML::RARA* expression in the *Ctsg-PML::RARA* knock-in mouse model. Bulk RNA-seq or ATAC-seq was performed on promyelocytes that were flow enriched from the bone marrow of *Ctsg-PML::RARA* mice or WT littermates. (*A*) Volcano plot of expression changes between *Ctsg-PML::RARA* vs. WT promyelocytes by RNA-seq. (*B*) Heat maps of the 703 DEGs between *Ctsg-PML::RARA* vs. WT promyelocytes by RNA-seq (FDR ≤ 0.05 and fold-change ≥ 2). (*Left* plot) RNA-seq of *Ctsg-PML::RARA* or WT promyelocytes (*n* = 3, each). (*Right* plot) scRNA-seq of GFP+ cells retrovirally transduced with *PML::RARA*^WT^ vs. empty vector, passively plotted at the 703 DEGs from the *Left* plot. (*C*) Distribution of *Ctsg-PML::RARA* and WT accessible regions at various regions in the genome compared to that of the mm 10 reference genome (background). **P < 0.01. (*D*) Volcano plot of accessibility changes between *Ctsg-PML::RARA* vs. WT promyelocytes by ATAC-seq (FDR ≤ 0.05 and fold-change ≥ 2). (*E*, *Left* plot) Heat map of the 7,379 differentially accessible regions between *Ctsg-PML::RARA* vs. WT promyelocytes by ATAC-seq (*n* = 3, each). (*E*, *Right* plot) Motif enrichment using HOMER analysis (34) at regions that show increased or decreased accessibility. (*F*) Heat map of the 238 genes that were within 1 kb of the regions that showed increased accessibility. RNA-seq data from *Ctsg-PML::RARA* vs. WT promyelocytes is plotted.

Since retroviral expression of *PML::RARA* creates a population of cells with a unique transcriptional signature that has no comparable population in WT hematopoietic cells, we next orthogonally validated the transcriptional consequences of *PML::RARA* in vivo, using nontransduced cells expressing *PML::RARA*. To do this, we performed bulk RNA-seq on flow-sorted promyelocytes from three 8- to 12-wk-old, littermate-matched *Ctsg-PML::RARA* vs. WT mice (2 pairs of males and 1 pair of females). This approach allowed us to compare the expression profiles of cells at the same stage of differentiation, and led to the identification of 703 DEGs (356 up-regulated, including *Gata2*; 347 down-regulated; FDR ≤ 0.05 and fold-change ≥ 2) (Fig. 3 *A* and *B* and Dataset S7). 150 DEGs were coordinately dysregulated (67 up-regulated and 83 down-regulated) by *PML::RARA* in both mouse RNA-seq models (Fig. 3 *B*, *Right*).

Using the same *Ctsg-PML::RARA* model, we evaluated the effects of *PML::RARA* expression on DNA accessibility in chromatin: We performed assay for transposase-accessible chromatin sequencing (ATAC-seq) on flow-enriched promyelocytes from littermate-matched, 8 to 12-wk-old *Ctsg-PML::RARA* or WT mice (1 pair of males and 2 pairs of females). ATAC-seq peaks were identified at 101,442 and 98,093 regions in WT and *Ctsg-PML::RARA* promyelocytes, respectively. ATAC-seq peaks were enriched at promoters, TSSs, and enhancers (Fig. 3*C*). 82,070 (>80%) were shared by both genotypes (i.e., they overlapped by at least one base pair). Although accessibility was very similar between *Ctsg-PML::RARA* and WT promyelocytes, *Ctsg-PML::RARA* promyelocytes had alterations in DNA accessibility at 7,379 of the 82,070 shared peaks (4,220 regions with increased accessibility, and 3,159 decreased accessibility) (FDR ≤ 0.05 and fold-change ≥ 1.5; Fig. 3 *D* and *E* and Dataset S8). An additional 5,753 regions showed genotype-specific accessibility; 3,593 regions were accessible only in *Ctsg-PML::RARA* promyelocytes and 2,160 regions were accessible only in WT promyelocytes (fold-change ≥ 1.5; Datasets S9 and S10). By integrating the expression and chromatin accessibility data, we observed that these changes in accessibility were strongly associated with coordinate changes in RNA expression in the same *Ctsg*-*PML::RARA* model (Fig. 3*F*).

To determine whether chromatin structure perturbations caused by *PML::RARA* expression are dependent upon its binding to DNA, we performed ATAC-seq on lineage-depleted mouse bone marrow cells 7 d following transduction with *PML::RARA*^WT^, *PML::RARA*^C88A^, or empty vector MSCV-IRES-GFP retroviruses. Comparison of *PML::RARA*^WT^ vs. *PML::RARA*^C88A^ transduced cells revealed that 3,038 (41.2%) of the *Ctsg-PML::RARA* vs. WT promyelocyte differentially accessible regions were coordinately regulated by *PML::RARA*^WT^ in a DNA binding–dependent manner. 2,335 regions were increased in accessibility, including those near the *Gata2* and *Mmp2* genes, and 703 regions were decreased (*SI Appendix*, Fig. S8 *A*–*D* and Datasets S11 and S12). In sum, these data show that *PML::RARA* expression leads to bidirectional changes in the DNA accessibility of a large number of genomic regions, many of which are dependent upon PML::RARA binding to DNA; many of these changes in DNA accessibility have consequences for gene expression.

### PML::RARA Binding Leads to Changes in Chromatin Accessibility, Including the GATA2 Locus.

**Fig. 4.**
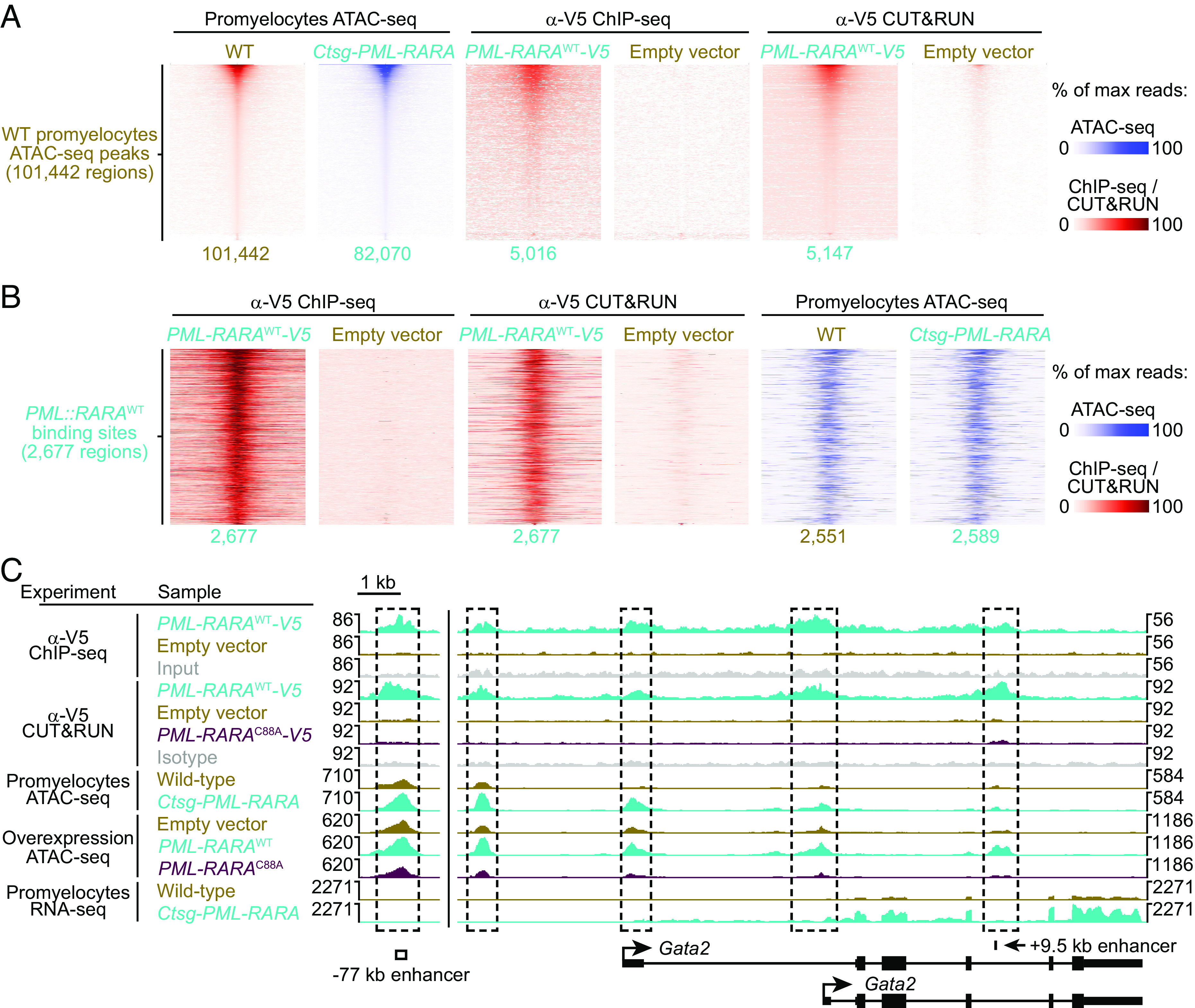
Integration of PML::RARA genomic binding and accessibility data. (*A*) Tornado plots of the 101,442 DNA accessible regions in WT promyelocytes by ATAC-seq plotted along the *Y*-axis. ATAC-seq, anti-V5 ChIP-seq, and anti-V5 CUT&RUN are passively plotted at the WT promyelocyte accessible regions. 82,070 of the 101,442 accessible regions in the WT promyelocytes are also accessible in *Ctsg-PML::RARA* promyelocytes. 5,016 and 5,147 of the 101,442 WT promyelocyte accessible regions are bound by PML::RARA^WT^ by anti-V5 ChIP-seq, or CUT&RUN, respectively. (*B*) Tornado plots of the 2,677 PML::RARA^WT^ binding sites. Anti-V5 ChIP-seq, anti-V5 CUT&RUN, and ATAC-seq are passively plotted. 2,551 and 2,589 of the 2,677 PML::RARA^WT^ binding sites are accessible in WT and *Ctsg-PML::RARA* promyelocytes by ATAC-seq, respectively. (*C*) Genome browser tracks for the *Gata2* locus, including the −77 kb enhancer (45, 46), and +9.5 kb *Gata2* intronic enhancer (47). The *Y*-axis is the mean read depth per bp.

We next wanted to define the relationships between PML::RARA binding and DNA accessibility. Integration of these datasets revealed that 95.1% of PML::RARA^WT^ binding sites directly overlapped with ATAC-seq peaks that were found in both WT and *Ctsg-PML::RARA* promyelocytes (Fig. 4 *A*–*C*). When combined with the motif analysis, this suggests that PML::RARA binds to RARE motifs that are found in regions of accessible chromatin in WT promyelocytes (Fig. 1*E*). This also suggests that these regions are “open” prior to PML::RARA expression, perhaps because of the normal binding of one or more “pioneer” transcription factors to these regions in WT hematopoietic cells. This hypothesis is supported by the fact that only 205 PML::RARA^WT^ binding events (7.5%) induced changes in DNA accessibility (186 increased and 29 decreased) (FDR ≤ 0.05 and fold-change ≥ 1.5) (Fig. 4*B* and *SI Appendix*, Fig. S8*E*). Consistent with what has been reported for other transcription factors (43, 44), the vast majority of PML::RARA^WT^ binding events do not appear to change DNA accessibility. These data suggest that most of the changes in DNA accessibility driven by PML::RARA expression are indirect.

To identify potential factors that may mediate PML::RARA-induced changes in DNA accessibility, we performed motif analysis on the differentially accessible regions between *Ctsg-PML::RARA* vs. WT promyelocytes. Specific motifs were enriched at sites that showed increased accessibility with *PML::RARA* expression in promyelocytes, including motifs for GATA (2,130 regions, *P* < 1e-294) and CTCF (275 regions, *P* < 1e-17) (Fig. 3*E*). GATA motifs were similarly enriched at sites with increased in accessibility following *PML::RARA* expression in mouse and human hematopoietic progenitors retrovirally transduced with *PML::RARA*^WT^ (*SI Appendix*, Figs. S8*B* and S9 *A* and *B*), and was the only motif that was preferentially enriched in the regions with increased DNA accessibility in all ATAC-seq models examined. This suggests that one or more GATA factors bind at thousands of loci, acting as pioneer factors that can increase DNA accessibility at sites that are permissive for PML::RARA binding.

We previously determined that *GATA2* is highly expressed in cells that express *PML::RARA* (12). Our ChIP-seq data further demonstrated that PML::RARA binds to the *GATA2* promoter and *GATA2* distal upstream enhancer, a finding that is associated with increased DNA accessibility at these sites in human and mouse hematopoietic cells (Fig. 4*C* and *SI Appendix*, Fig. S10*A*). The increased accessibility coincided with high levels of *GATA2* following *PML::RARA* expression (Fig. 4*C* and *SI Appendix*, Fig. S10 *B*–*D*). Together, these data suggest that PML::RARA may directly activate the expression of *GATA2*; in turn, GATA2 may mediate many of the changes in DNA accessibility following *PML::RARA* expression.

### GATA2 and PML::RARA Bind to Contiguous Genomic Regions to Cooperatively Regulate DNA Accessibility and Transcription.

**Fig. 5.**
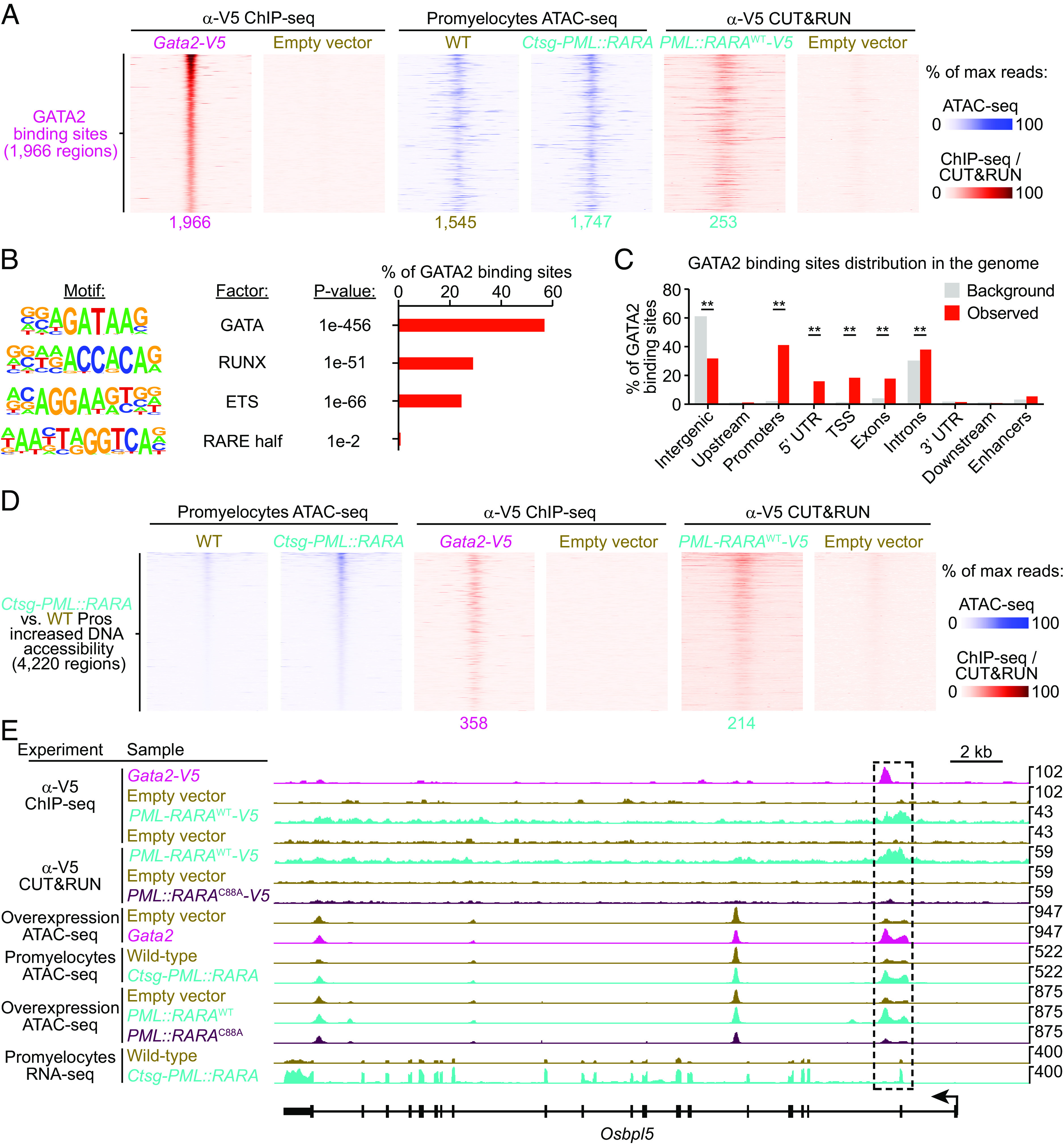
GATA2 and PML::RARA bind to contiguous genomic regions where they cooperatively regulate chromatin accessibility and transcription. Lineage-depleted WT mouse bone marrow cells were transduced with retroviruses containing *Gata2-V5* or an empty vector. Four days after transduction, anti-V5 ChIP-seq was performed. (*A*) Tornado plots of the 1,966 GATA2 binding sites determined by anti-V5 ChIP-seq in cells transduced with *Gata2-V5* plotted along the *Y*-axis. ATAC-seq and anti-V5 CUT&RUN are passively plotted. 1,545 and 1,747 of the 1,966 GATA2 binding sites are accessible in WT and *Ctsg-PML::RARA* promyelocytes, respectively. 253 of the 1,966 GATA2 binding sites also bound by PML::RARA^WT^. (*B*) Motif enrichment at GATA2 binding sites by HOMER analysis (34). (*C*) Distribution of GATA2 binding sites at various regions in the genome compared to that of the mm10 reference genome (background). **P < 0.01. (*D*) Tornado plots of the 4,220 regions that showed increased DNA accessibility in *Ctsg-PML::RARA* vs. WT promyelocytes by ATAC-seq plotted along the *Y*-axis. Anti-V5 ChIP-seq and anti-V5 CUT&RUN are passively plotted. 358 and 214 of the 4,220 regions that showed increased DNA accessibility in *Ctsg-PML::RARA* vs. WT promyelocytes were bound by GATA2 and PML::RARA^WT^ respectively; 52 were bound by both GATA2 and PML::RARA^WT^. (*E*) Genome browser tracks for the *Osbpl5* locus.

To determine whether GATA2 could mediate many of the epigenetic changes associated with *PML::RARA* expression, and to identify the binding sites of GATA2 in primary hematopoietic cells, we generated a V5-tagged *Gata2* (*Gata2-V5*) MSCV-IRES-GFP retroviral construct. To verify that the V5 tag did not affect the function of *Gata2*, we transduced bone marrow cells from *Ctsg-PML::RARA* mice with *Gata2-V5,* untagged *Gata2*, or an empty vector, and performed serial replating. Robust expression of GATA2 protein was verified 4 d following transduction (*SI Appendix*, Fig. S11*A*). Similar to untagged *Gata2* (12), we found that *Gata2-V5* was selected against following 3 wk of serial replating (*SI Appendix*, Fig. S11*B*). Anti-V5 ChIP-seq in lineage-depleted WT mouse bone marrow cells transduced with *Gata2-V5* identified 1,966 GATA2 binding sites (Fig. 5*A*). As expected, consensus GATA binding motifs were found at 1,118 (56.9%) of GATA2 binding sites (Fig. 5*B*). GATA2 binding was enriched at promoters and TSSs (41.2%, and 18.4% of sites, respectively) (Fig. 5*C*), including several that are near *Gata2* regulated genes, including *Gata1, Zfpm1, Tal1, Spi1 (Pu.1),* and *Il4* (48–50). GATA2 binding occurred within 1 kb of 1,447 genes, which were enriched for pathways involving HDAC-mediated signaling, inflammation, Hedgehog signaling, Wnt Signaling, cytokine and chemokine signaling, and acute myeloid leukemia, among others (*SI Appendix*, Fig. S11*C* and Dataset S13).

We next evaluated how GATA2 binding influences DNA accessibility in *PML::RARA* expressing cells. Integration with the promyelocyte ATAC-seq dataset revealed that regions that showed increased DNA accessibility following *PML::RARA* expression displayed enrichment of GATA2 binding (*P* < 0.01 by permutation test) (Fig. 5*D*). This suggests that GATA2 is acting as a pioneer factor at such sites. In addition, we found that GATA2 and PML::RARA bound in close proximity (with binding sites that overlapped by at least 1 bp) at 253 regions in the genome, including the *Osbpl5* locus (Fig. 5 *A* and *E* and *SI Appendix*, Fig. S11*D*), as an example that has been implicated in AML biology (51, 52). Motif analysis at these co-occupied regions revealed that the average distance between RARE half motifs and GATA motifs was ~85 bp (*SI Appendix*, Fig. S11*E*), suggesting that GATA2 and PML::RARA may cooperatively bind to such shared target loci, rather than competing for binding at overlapping target sequences. Moreover, RARE and GATA motifs are nonrandomly distributed in the genome, with an average spacing of ~565 bp in the mouse genome and ~561 bp in the human genome—a distance that is closer than expected by chance (*P* < 0.01 by permutation test) (*SI Appendix*, Fig. S11 *F* and *G*). These data suggest that there may be a physiologic RAR-GATA transcriptional network that is hijacked by PML::RARA.

To determine whether *Gata2* could be causing some of the epigenetic changes associated with *PML::RARA* expression, we performed ATAC-seq on WT lineage-depleted mouse bone marrow cells 4 d after retroviral transduction with *Gata2* or an empty vector. *Gata2* expression led to changes in DNA accessibility at 39,873 regions (FDR ≤ 0.05 and fold-change ≥ 1.5; 20,393 increased, 19,480 decreased) (*SI Appendix*, Fig. S12 *A* and *B* and Dataset S14). 2,873 (68.1%) of the regions that showed increased expression following *PML::RARA* expression in promyelocytes showed coordinate increases in DNA accessibility following *Gata2* expression in the absence of *PML::RARA* (*SI Appendix*, Fig. S12 *C*–*E*). Additionally, 341 sites showed GATA2 binding and coordinate increases in DNA accessibility following expression of *PML::RARA* in promyelocytes and *Gata2* in WT hematopoietic cells—these sites may be directly “opened” by *Gata2* following positive regulation of *Gata2* by *PML::RARA*.

To determine whether *Gata2* could also influence the transcriptional changes that follow *PML::RARA* expression, we retrovirally transduced lineage-depleted WT mouse bone marrow with *Gata2* MSCV or an empty vector, and then performed scRNA-seq on the transduced cells 4 d following transduction. *Gata2* overexpression led to 1,664 DEGs (944 up-regulated, 720 down-regulated; FDR ≤ 0.05 and fold-change ≥ 2) (*SI Appendix*, Fig. S13*A* and Dataset S15). This analysis revealed that 263 of the *Ctsg-PML::RARA* vs. WT promyelocyte DEGs were coordinately regulated by *Gata2* (147 up-regulated and 116 down-regulated) (*SI Appendix*, Fig. S13*B*). To identify *PML::RARA*^WT^ DEGs that are dependent upon *Gata2*, we inactivated *Gata2* using CRISPR/Cas9 in *Ctsg-PML::RARA* hematopoietic cells (12) and then performed scRNA-seq. *Gata2* deficiency produced 863 DEGs (501 up-regulated, 362 down-regulated with *Gata2* deficiency; FDR ≤ 0.05 and fold-change ≥ 2) (*SI Appendix*, Fig. S13*C* and Dataset S16). We found that 106 of the *Ctsg-PML::RARA* DEGs were dependent upon *Gata2* (76 up-regulated by *PML::RARA* and down-regulated by *Gata2* deficiency, and 30 down-regulated) (*SI Appendix*, Fig. S13*B*). These *Gata2*-dependent DEGs included *Hgf, Pdgfrb, Hdc, Igfbp7,* and *Lgals3bp*, all of which have been implicated in cancer pathogenesis and/or hematopoiesis (53–57); some have previously been described as being regulated by *PML::RARA* (including *Hgf*, *Pdgfrb,* and *Hdc*) (12, 58). These data therefore suggest that *Gata2* may mediate a substantial portion of the epigenetic and transcriptional changes that accompany *PML::RARA* expression.

### PML::RARA and GATA2 Proteins Interact via Binding to DNA.

**Fig. 6.**
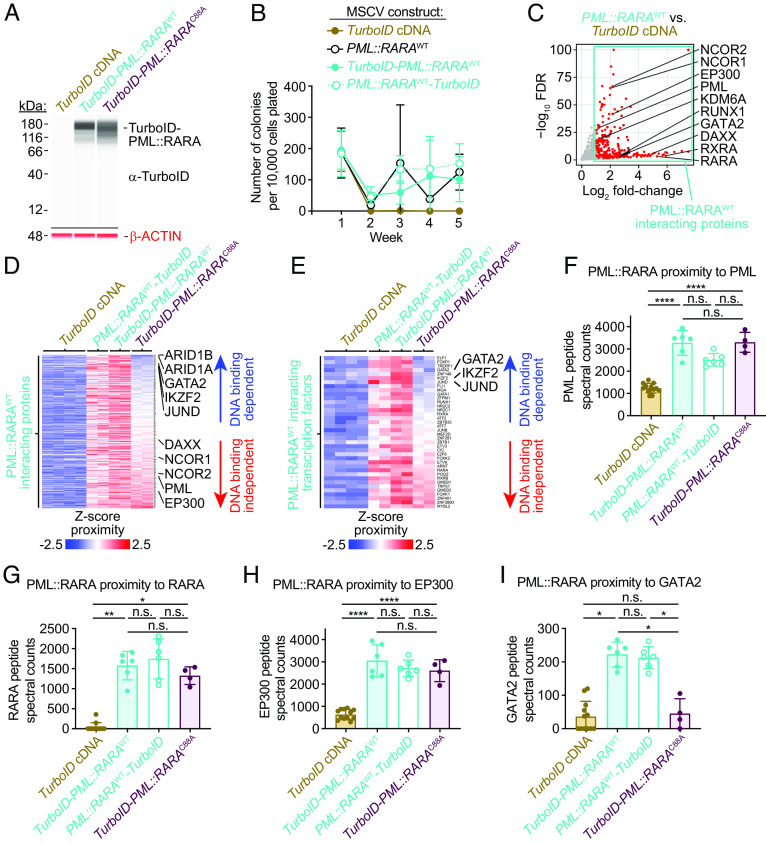
PML::RARA interacts with GATA2 and several other proteins via binding to DNA. Proximity labeling assays in which lineage-depleted mouse bone marrow cells were transduced with retroviruses containing *TurboID-PML::RARA*^WT^, *PML::RARA*^WT^*-TurboID*, *TurboID-PML::RARA*^C88A^*, TurboID* cDNA alone, or *PML::RARA*^WT^. Transduced cells were labeled with biotin, and interacting proteins were enriched with streptavidin bead pull-downs and identified by mass spectrometry.(*A*) Anti-TurboID (*Top* blot) and anti-beta ACTIN (*Bottom* blot) western blot analysis on the day of the biotin labeling and bead pull-downs. (*B*) Colony counts from serial replating assays (*n* = 3, each). (*C*) Volcano plot of the proteins that showed increased interaction with PML::RARA^WT^ (both *PML::RARA*^WT^*-TurboID* and *TurboID-PML::RARA*^WT^) compared to TurboID cDNA alone. (*D*) Heat map of the PML::RARA^WT^ interacting proteins ordered by the fold change in interaction with PML::RARA^WT^ (*PML::RARA*^WT^*-TurboID* and *TurboID-PML::RARA*^WT^), compared to the interaction with TurboID-PML::RARA^C88A^. Proteins labeled with TurboID alone are passively plotted. Each column represents an independent biological replicate and is representative of a total of *n* = 8 for TurboID cDNA, *n* = 4 for *PML::RARA*^WT^*-TurboID*, *n* = 4 for *TurboID-PML::RARA*^WT^, and *n* = 4 for *TurboID-PML::RARA*^C88A^ (see *SI Appendix*, Fig. S14 for the remaining replicates). (*E*) Heat map of the transcription factors interacting with PML::RARA^WT^, ordered by the fold change in interaction with PML::RARA^WT^ (*PML::RARA*^WT^*-TurboID* and *TurboID-PML::RARA*^WT^) compared the interaction with TurboID-PML::RARA^C88A^. TurboID cDNA alone is passively plotted. (*F–I*) Normalized peptide spectral counts of PML (*F*), RARA (*G*), EP300 (*H*), and GATA2 (*I*) following proximity labeling. ****FDR ≤ 1e-24, **FDR ≤ 0.01, *FDR ≤ 0.05, and n.s. = not significant by edgeR (59).

Our data suggest that PML::RARA and GATA2 bind in close proximity to one another at hundreds of sites in the genome. To determine whether PML::RARA binds in close proximity to GATA2, and whether this interaction might be mediated by the DNA template where both proteins bind, we performed proximity labeling experiments. We transduced lineage-depleted mouse bone marrow cells with MSCV-IRES-GFP-based retroviruses containing an enhanced biotin ligase (“TurboID”) fused to either the N terminus or C terminus of *PML::RARA*^WT^ (*TurboID-PML::RARA*^WT^ and *PML::RARA*^WT^*-TurboID,* respectively), *TurboID-PML::RARA*^C88A^, or the *TurboID* cDNA alone (Fig. 6*A*). A flexible Glycine/Serine linker placed between TurboID and PML::RARA allows TurboID to freely rotate, and biotinylate proteins within 10 Å of the TurboID-PML::RARA fusion protein. We independently showed that addition of *TurboID* to *PML::RARA* did not affect the ability of *PML::RARA* to cause the aberrant serial replating characteristic of this fusion (Fig. 6*B*). In addition, both *TurboID-PML::RARA*^WT^ and *PML::RARA*^WT^*-TurboID* disrupted PML nuclear body formation into “microspeckles”, another canonical feature of *PML::RARA* expression (*SI Appendix*, Fig. S14 *A* and *B*). In agreement with a previous report (19), *TurboID-PML::RARA*^C88A^ also induced microspeckles, suggesting that this activity may not be dependent upon PML::RARA binding to DNA (*SI Appendix*, Fig. S14 *A* and *B*).

To identify the protein “interactome” of PML::RARA in mouse hematopoietic cells, we performed streptavidin bead pull-downs and mass spectrometry on biotin-treated cell lysates expressing the *PML:RARA-TurboID* constructs. This led to the identification of 268 proteins that interact with PML::RARA^WT^ [compared to cells transduced with a *TurboID* cDNA alone; FDR ≤ 0.05 and fold-change ≥ 2 using edgeR (59)] (Fig. 6*C* and Dataset S17). Importantly, *TurboID-PML::RARA*^WT^*, PML::RARA*^WT^*-TurboID,* and *TurboID-PML::RARA*^C88A^ were associated with “self” pulldown interactions with PML and RARA, as expected, and interactions with many previously identified binding partners, including EP300, NCOR1, NCOR2, and DAXX (among others) (24–30) (Fig. 6 *C*–*I* and *SI Appendix*, Fig. S14*C*). PML::RARA interacted with the UTY protein (encoded by *Uty* on the Y chromosome) in cells from male but not female mice (*SI Appendix*, Fig. S14*D*), and interacted with the UTY paralog KDM6A/UTX (encoded by *Kdm6a* on the X chromosome) in cells from both male and female mice (*SI Appendix*, Fig. S14*E*). We found that 34 PML::RARA^WT^ interactions were dependent upon PML::RARA binding to DNA, since these interactions did not occur with TurboID-PML::RARA^C88A^; these included interactions with the SWI/SNF components ARID1A and ARID1B, and several transcription factors, including GATA2, JUND, and the ETS family members IKZF2 and ELF1 (FDR ≤ 0.05 and fold-change ≥ 2 by edgeR) (Fig. 6 *D*, *E*, and *I*, *SI Appendix*, Fig. S14 *C* and *F*, and Dataset S18). These data corroborate our ChIP-seq data, which showed that GATA, JUN, and ETS motifs are highly enriched within PML::RARA^WT^ binding sites. The interaction of PML::RARA with GATA2 has also been documented in coimmunoprecipitation assays (30), providing further evidence of this interaction. PML::RARA binds DNA in close proximity to these transcription factors, suggesting that DNA binding by PML::RARA also enhances these protein–protein interactions.

### Gata2 Is Required for PML::RARA to Establish a Self-Renewal Program.

**Fig. 7.**
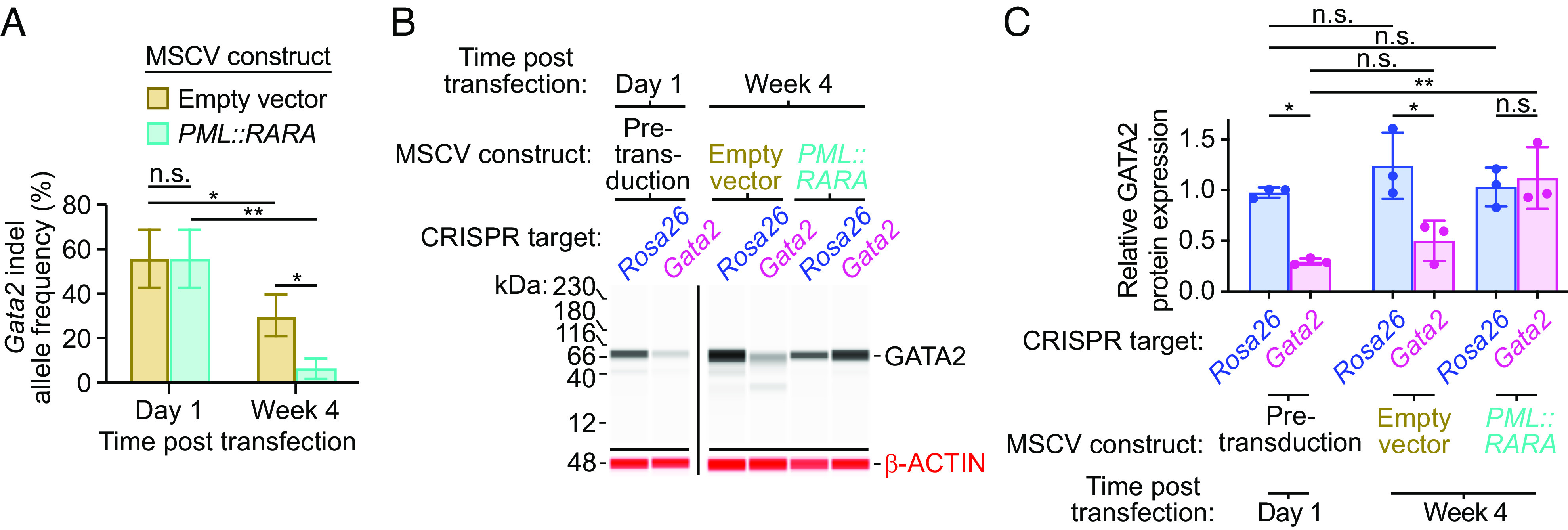
Serial replating assays of *Gata2* CRISPR/Cas9 genome-edited cells before or after *PML::RARA* expression. Lineage-depleted bone marrow cells from *Cas9-GFP* mice were electroporated with *Gata2* or *Rosa26* guide RNAs, followed by transduction with *PML::RARA* or empty vector retroviruses 3 d later. Cells were then serially replated in MethoCult^®^ M3434. (*A*) *Gata2* insertion/deletion (indel) frequency by digital sequencing at 1 d and 4 wk following transfection with CRISPR/Cas9 guide RNAs targeting the *Gata2* locus. (*B*) Anti-GATA2 (*Top* blots) or anti-beta-ACTIN (*Bottom* blots) western blot analysis in cells at day 1 or week 4 following transfection with CRISPR/Cas9 guide RNAs and subsequent transduction with *PML::RARA* or empty MSCV retroviruses (n = 3, each). (*C*) Quantification of the relative GATA2 to beta-ACTIN protein expression from (*B*) (n = 3, each). **P < 0.01, *P < 0.05, and n.s. = not significant by two-way ANOVA.

The data above suggested that *Gata2* inactivation prior to *PML::RARA* expression may prevent its ability to cause self-renewal. To test this, we inactivated *Gata2* [or deleted a portion of *Rosa26* intron 1 as a negative control (12)] using CRISPR/Cas9 genome editing in lineage-depleted bone marrow from *Cas9-GFP* mice. The average initial targeting efficiency of *Gata2* was 56.5% 1 d following guide RNA transfection (Fig. 7*A*), typically resulting in homozygous deletions (12). The remaining cells that were WT for *Gata2* should maintain their ability to aberrantly self-renew upon expression of *PML::RARA*. We confirmed by western blotting that GATA2 protein levels were reduced in *Gata2* vs. *Rosa26*-targeted cells the day after transfection (Fig. 7 *B* and *C*). The transfected cells were then transduced with *PML::RARA* or empty vector MSCV-IRES-Thy1.1 retroviruses 3 d after transfection and serially replated in MethoCult M3434. After 4 wk, the knockout *Gata2* allele frequency in *PML::RARA* transduced cells had decreased by 8.31-fold, to an average of 6.80% (Fig. 7*A*; *P* = 0.0012 by two-way ANOVA), and the *Gata2*-targeted cells expressed as much GATA2 protein as the *Rosa26*-targeted cells (Fig. 7 *B* and *C*). This suggests that the *Gata2*-deficient, *PML::RARA*-expressing cells were not able to aberrantly self-renew. In contrast, the frequency of knockout *Gata2* alleles was 30.25% in cells transduced with an empty vector—suggesting that *Gata2* is important for PML::RARA-induced self-renewal, but not as essential for the growth of WT progenitors in this context. We previously established that inactivation of *Gata2 after PML::RARA* expression leads to an increased frequency of knockout *Gata2* alleles within 4 wk (12); in that context, *PML::RARA* establishes the aberrant self-renewal phenotype, and *Gata2* appears to act as a tumor suppressor to limit the proliferative stress caused by *PML::RARA*. In sum, these data suggest that *Gata2* is necessary for *PML::RARA* to initiate its aberrant self-renewal program; once established, *Gata2* is dispensable for self-renewal.

## Discussion

In this study, we identified the epigenetic and transcriptional consequences of *PML::RARA* expression in primary mouse and human hematopoietic cells. Surprisingly, we found that most of the genomic DNA binding sites of PML::RARA were accessible even in WT myeloid progenitors and that GATA2 is also bound near many of these sites; the two proteins interact, and their interaction requires PML::RARA to be bound to DNA. PML::RARA binds to the distal upstream *GATA2* enhancer (and to multiple intragenic *GATA2* regions), increases the DNA accessibility of regions within the *GATA2* locus, and leads to increased *GATA2* expression in primary human and mouse hematopoietic cells. *Gata2* overexpression leads to increased DNA accessibility at many PML::RARA binding sites, and at many regions that are opened following *PML::RARA* expression. GATA2 in turn mediates many of the transcriptional reprogramming events that are induced by *PML::RARA*. In fact, the ability of *PML::RARA* to induce self-renewal is dependent upon *Gata2* itself. Our data suggest a model where *PML::RARA* positively regulates *Gata2* expression, and GATA2 then cooperates with PML::RARA through DNA-templated protein–protein interactions to reprogram myeloid progenitors (*SI Appendix*, Fig. S1).

Previous studies have used a variety of approaches to identify the genomic binding sites of PML::RARA in cell lines (PR-9 and NB4) (21–23) and two primary human APL samples (22). NB4 cells are derived from an APL patient, and express a bcr1 *PML::RARA* mRNA from a classical t(15;17) translocation; however, these cells also have *TP53* mutations and are aneuploid (both are extremely rare in primary APL samples and probably acquired during immortalization) (60–63). PR-9 cells were made by stably integrating a Zinc-inducible *PML::RARA* cDNA into the promonocyte U937 cell line, which was derived from a patient with a histiocytic lymphoma (20). Because of these caveats and the lack of consensus between these studies, we therefore used a V5 tagging strategy to identify PML::RARA binding sites in the chromatin of both primary mouse and human hematopoietic cells.

Due to these differing approaches, it is perhaps not surprising that the PML::RARA binding sites identified in this study were different from the previously reported ones (*SI Appendix*, Fig. S1*B*). Many previously unidentified binding sites were detected: A total of 1,001 (48.5%) sites were not found in any of the previous studies, including prominent ones at the *GATA2* promoter and within the *GATA2* gene (Fig. 4*C* and *SI Appendix*, Fig. S10*A*). Analysis of concordance among studies revealed that only 50 sites were common to all four. Only 318 sites identified here were in common with the Wang et al. study (21); 203 sites were in common with the Martens et al. study (22). The Tan et al. study (23) identified the most binding sites of all the studies (>6,000), and 975 were concordant with the sites identified here; this may be due to the antibody that allowed for direct detection of the PML::RARA fusion protein by ChIP-seq (but which is not absolutely specific for this fusion, as shown in *SI Appendix,* Fig. S2*C*). Clearly, both technical differences, and different cellular contexts, could account for many of these differences.

We have also shown that PML::RARA also interacts with several other hematopoietic transcription factors (including RUNX1, JUND, and IKZF2) and these interactions require the binding of PML::RARA to DNA. RARE half sites were enriched at these regions much more than RARE motifs with spacers, suggesting that PML::RARA binding to chromatin is stabilized by the proximal binding of GATA2 and/or other transcription factors (21). In normal hematopoietic cells, RARA may interact with these factors in a similar way, to facilitate precise temporal and myeloid developmental specificity (64, 65). RARA has in fact been shown to interact with GATA2 via the DNA binding domains of both proteins, suggesting that RARA and GATA2 cooperatively regulate normal hematopoiesis through DNA-templated interactions (66). PML::RARA may hijack this physiologic network to enforce its preleukemic program, synergizing with GATA2 in accessible DNA sites via one or more potential mechanisms: (1) GATA2 may act as a pioneer factor to make regions with RARE motifs more accessible to PML::RARA; (2) GATA2 and PML::RARA may synergize to dislodge nucleosomes at some shared sites [indeed, some pioneer factors have been shown to require other transcription factors to bind to some loci (65, 67–70)]; and/or (3) PML::RARA and GATA2 may synergize to recruit cofactors such as EP300 or HDAC3 to shared target loci (23, 71–74). This kind of transcriptional cooperativity has been described for other transcription factors (75); all three of these potential mechanisms may be DNA context-dependent, and all could be relevant for PML::RARA activity at specific binding sites in the genome.

Using proximity labeling, we identified many PML::RARA protein interactions, including several cofactors and chromatin modifiers that PML::RARA may recruit to specific loci to induce epigenetic regulation. Many of these interactions were independent of PML::RARA binding to DNA (e.g,. BCOR, JMJD1C, and ATRX), while others were DNA binding–dependent (e.g., ARID1A, ARID1B, and BRD8). The cofactors that were DNA binding–dependent may contact PML::RARA directly or indirectly via other transcription factors.

We found that the C88A mutation in PML::RARA disrupted the interaction of PML::RARA with GATA2. Although this finding could potentially be due to an altered ability of the mutant protein to interact with GATA2, we feel that this is unlikely for several reasons. First, C88A disrupts one of the eight critical cysteines in RARA that coordinate two zinc ions in the DNA binding domain of RARA (32). These cysteines have been extensively characterized (19, 31–33), and C88 is not known to have any functions other than mediating DNA binding. Mutation of two of the other zinc-coordinating cysteines in a similar zinc-finger domain in the glucocorticoid receptor has been shown to induce export of the that receptor from the nucleus to the cytoplasm (76). However, we did not observe any changes in cellular location between PML::RARA^WT^ and PML::RARA^C88A^—both were primarily located in the nucleus (*SI Appendix*, Fig. S14*A*). Therefore, differences in the protein interactions of PML::RARA^C88A^ and PML::RARA^WT^ are mostly likely to be due to a disruption in DNA binding by the C88A mutation.

The inactivation of *Gata2* before *PML::RARA* expression prevented the ability of *PML::RARA* to promote self-renewal in serial replating assays. This finding is similar to a previous study, which showed that heterozygous *Gata2* missense mutations occurring prior to the expression of *CBFB::MYH11* lead to longer leukemic latency (77). We previously demonstrated that inactivation of *Gata2* after *PML::RARA* expression leads to increased self-renewal, increased APL penetrance, and decreased APL latency (12). We similarly demonstrated that *Gata2* knockout after acquisition of *RUNX1-RUNX1T1* or biallelic *Cebpa* inactivation also leads to increased self-renewal (12). These findings are reinforced by the observation that acquired heterozygous *GATA2* mutations are associated with AML progression, and the remaining WT *GATA2* allele is nearly always epigenetically silenced (78–80). Further, patients with germline missense *GATA2* mutations have a mean onset of MDS/AML at age 40 (81, 82), suggesting that these mutations do not directly cause myeloid malignancies; inactivation of the second *GATA2* allele (by mutation or epigenetic silencing) appears to be necessary for AML development (78). Thus, some *Gata2* activity appears to be required early in leukemia initiation, to open key regions in chromatin. In this model, GATA2 and PML::RARA synergistically open such loci by binding to adjacent canonical binding motifs, with the subsequent recruitment of cofactors and chromatin modifiers. After a leukemic transcriptional program is established, however, *Gata2* acts as a tumor suppressor, performing its normal role to limit proliferative excess in hematopoietic progenitors; this is probably the reason that inactivation of the second allele promotes tumor progression (12). Together, these studies suggest that *GATA2* and other transcriptional networks are similarly hijacked by other mutations to initiate AML. Because these networks sometimes require protein–protein interactions for their function, some may be druggable with small molecules, potentially providing novel approaches for therapy. In addition, this study defines many of the epigenetic and transcriptional consequences of *PML::RARA* expression. Future studies will be required to determine which of these dysregulated gene(s) and/or protein interactions initiate the aberrant self-renewal and transformation of myeloid progenitors.

## Materials and Methods

### Human AML Samples.

Human AML samples were acquired as part of studies that were approved by the Washington University School of Medicine Human Research Protection Office. All the patients provided written informed consent that included explicit permission for genetic studies, under an Institutional Review Board–approved protocol (#201011766). All samples were prospectively anonymized and are considered “nonhuman” for that reason.

### Mice.

*Ctsg-PML::RARA* mice (7) were bred with *Rosa26-Cas9-GFP* mice (83) (Jackson Labs) to generate *Ctsg-PML::RARA^+/−^ x Rosa26-Cas9-GFP^+/−^ (Ctsg-PML::RARA x Cas9-GFP)* mice on a C57BL/6J background.

### ChIP-seq, CUT&RUN, ATAC-seq, and RNA-seq Analyses.

Details of the ChIP-seq, CUT&RUN, ATAC-seq, and RNA-seq analyses are presented in *SI Appendix, Supplementary Methods*.

### CRISPR/Cas9 Gene Editing.

Details of the CRISPR/Cas9 gene editing are presented in *SI Appendix, Supplementary Methods*.

### Proximity Labeling and Mass Spectrometry.

Details of the proximity labeling and mass spectrometry analyses are presented in *SI Appendix, Supplementary Methods*.

## Supplementary Material

Appendix 01 (PDF)

Dataset S01 (XLSX)

Dataset S02 (XLSX)

Dataset S03 (XLSX)

Dataset S04 (XLSX)

Dataset S05 (XLSX)

Dataset S06 (XLSX)

Dataset S07 (XLSX)

Dataset S08 (XLSX)

Dataset S09 (XLSX)

Dataset S10 (XLSX)

Dataset S11 (XLSX)

Dataset S12 (XLSX)

Dataset S13 (XLSX)

Dataset S14 (XLSX)

Dataset S15 (XLSX)

Dataset S16 (XLSX)

Dataset S17 (XLSX)

Dataset S18 (XLSX)

## Data Availability

All sequencing data for the mouse studies were deposited to the Sequence Read Archive at the NCBI, PRJNA1097608 (84); promyelocyte RNA-seq data can also be visualized using an interactive application at: https://aplpros.leylab.org/. Sequencing data for the human studies were deposited to the database of Genotypes and Phenotypes (dbGaP) at the NCBI, phs000159 (85). TurboID mass spectrometry data were deposited to the ProteomeXchange, PXD044816 (86), and can be visualized using an interactive application at https://pmlrara-turboid.leylab.org/.
