# Supplementary material for: PML::RARA and GATA2 proteins interact via DNA templates to induce aberrant self-renewal in mouse and human hematopoietic cells
Source: Proc Natl Acad Sci U S A. 2024 Apr 22;121(18):e2317690121. doi: 10.1073/pnas.2317690121 (PMC11067031; doi:10.1073/pnas.2317690121)
Supplement: Supplementary file 1 — Appendix 01 (PDF) [file pnas.2317690121.sapp.pdf]

PNAS

Supporting Information for

PML::RARA and GATA2 proteins interact via DNA templates to induce aberrant self-renewal in mouse and human hematopoietic cells

Casey D.S. Katerndahl, Olivia R.S. Rogers, Ryan A. Day, Ziheng Xu, Nichole M. Helton, Sai Mukund Ramakrishnan, Christopher A. Miller, and Timothy J. Ley*

Section of Stem Cell Biology, Division of Oncology, Department of Internal Medicine

Washington University School of Medicine, St. Louis, MO 63110

Email: timley@wustl.edu

This PDF file includes:

Supplementary Methods
Figures S1 to S14
Tables S1 to S2
Legends for Datasets S1 to S18
SI References

Other supporting materials for this manuscript include the following:

Datasets S1 to S18

Supplementary Methods

Cell Lines

NB4 and K562 cells were purchased from ATCC. MOLM-13 and OCI-AML3 cells were purchased from the Leibniz Institute DSMZ.

Retroviral constructs

A V5 epitope tag was cloned in-frame on the 5' end of *PML::RARA*^{WT} or *PML::RARA*^{C88A}, or the 3' end of *PML::RARA*^{WT}*, PML::RARA*^{C88A}, or *Gata2* within MSCV based retroviral constructs. The MSCV-IRES-Thy1.1 backbone was a kind gift from the Michael Farrar lab (1). The Thy1.1 in these constructs only contains the extracellular and transmembrane domains, without an intracellular domain, so that Thy1.1 cannot transmit signals into cells. The TurboID reference sequence was obtained from Addgene plasmid #107171. A glycine-serine linker in-frame with TurboID was synthesized by IDT and subcloned into MSCV-IRES-GFP by Genewiz/Azenta. TurboID was cloned in-frame on the 5' end of *PML::RARA*^{WT} or *PML::RARA*^{C88A}, or the 3' end of *PML::RARA*^{WT} with a flexible serine/glycine linker in between *TurboID* and PML::RARA. Sequence was confirmed for all plasmids using Sanger sequencing of the insert, or whole-plasmid sequencing by Plasmidsaurus. N- and C-terminal cloning plasmids were deposited in Addgene (plasmid IDs 207957 and 207958). The sequences of the V5 tag and TurboID linker can be found in SI Appendix, Table S2.

Promyelocyte isolation

Promyelocytes were purified from bone marrow from *Ctsg-PML::RARA* or WT littermate mice using flow cytometry as previously described (2).

Human CD34+ cell isolation

Healthy human cord blood samples were deidentified prior to use in the study. Fresh cord blood

was diluted with equal volumes of room temperature phosphate buffer saline (PBS; Gibco 14040133), layered on top of Ficoll (Cytiva), and spun at 665g for 25 min at 19°C with no brake. Cells at the interphase were transferred and diluted 8-fold with cold PBS, and then spun at 300 g at 4°C for 7 minutes. Cells were resuspended in cold 1x human RBC buffer (1.5 mM NH_4Cl, 10 mM EDTA at pH7.4, and 97.6 μM $NaHCO_3$), incubated for 5 minutes on ice, spun at 300 g at 4°C for 6 minutes. Cells were resuspended in MACS buffer (PBS, 2 mM EDTA 0.5% BSA), and stained with Miltenyi CD34 microbeads (Cat. #: 130-046-702) according to manufactures instructions and purified with a Miltenyi AutoMACS using the program POSSELDS. Cells were resuspended and cultured in human hematopoietic progenitor cell media (DMEM, 15% FBS, 50 μM beta-mercaptoethanol, 1x penicillin-streptomycin [Gibco 15140122], 200 ng/ml SCF, 20 ng/ml FLT3L, 20 ng/ml TPO, 20 ng/ml IL-3, 40 ng/ml IL-6 [all cytokines from Peprotech]) at 37C with 5% CO_2.

Retroviral transductions

Bone marrow from C57Bl/6 mice was lineage-depleted and grown in mouse bone marrow media (RPMI, 15% fetal bovine serum [Atlas Biologicals], 1% penicillin-streptomycin [Gibco], 100 ng/mL mouse [m] SCF, 50 ng/mL mFLT3L, 6 ng/mL mIL-3, and 10 ng/mL mTPO [Peptrotech]) before and after transduction as previously described (3). Lineage-depleted mouse bone marrow cells were then transduced with MSCV-IRES-Thy1.1 retroviruses containing *V5-PML::RARA*^{WT}, *V5-PML::RARA*^{C88A}, *PML::RARA*^{WT}*-V5*, *PML::RARA*^{C88A}*-V5*, or an empty vector (MSCV backbone alone), or MSCV-IRES-GFP retroviruses containing *PML::RARA*^{WT}, *PML::RARA*^{C88A}, *Gata2-V5*, *TurboID-PML::RARA*^{WT}, *TurboID-PML::RARA*^{C88A}, *PML::RARA*^{WT}*-TurboID*, *TurboID* cDNA alone, or an empty vector as previously described (3). Human CD34 enriched cells were transduced with MSCV-IRES-GFP retroviruses containing *V5-PML::RARA*^{WT}, *V5-PML::RARA*^{C88A}, *PML::RARA*^{WT}, *PML::RARA*^{C88A}, or an empty vector as

previously described (3). Retroviruses were packaged with either pVSV-G or pAmpho helper plasmids (Clonetech) for the transduction of mouse and human cells respectively.

ChIP-seq and CUT&RUN

To identify the binding sites of PML::RARA^{WT}, lineage-depleted mouse bone marrow cells were transduced with *PML-RARA*^{WT}*-V5*, *PML-RARA*^{C88A}*-V5*, or empty MSCV-IRES-Thy1.1 retroviruses (1). Transduced Thy1.1+ cells were purified two days after the first transduction using Miltenyi mouse CD90.1 micro beads (Cat. #: 130-121-273) and an AutoMACS using the program POSSELS. Cells resuspended in room temperature PBS and treated with 1 mM Diisopropyl fluorophosphate (DFP) for 15 minutes, then washed 3 times with cold PBS, fixed with 1% formaldehyde, and washed 2 times with PBS. ChIP-seq was performed with SimpleChIP kit (Cell Signaling). Nuclei were lysed using a Covaris ME220 (100 seconds, peak power 70, 5% duty factor, 200 cycles/burst). 6 million cells, 1.5 uL of Micrococcal nuclease, and 3 ug of anti-V5 antibody (Abcam ab9116) were used per immunoprecipitation. GATA2 binding sites were identified using the same ChIP-seq method except lineage-depleted mouse bone marrow cells were transduced with *Gata2-V5* or empty MSCV-IRES-GFP retroviruses; GFP+ cells were flow purified 4 days following transduction and anti-V5 ChIP-seq was performed without DFP treatment. Input DNA was isolated and sequenced from lysed nuclei as a negative control. DNA eluted from Protein G beads was treated with RNase for 7 minutes at 37°C, then treated with proteinase K + NaCl at 55°C for 1 hour and then 65°C for 2 hours. DNA was then purified by Phenol Chloroform extraction using MaXtract tubes (Qiagen) as previously described. DNA was resuspended in 51 uL of buffer EB (Qiagen) (4). CUT&RUN was performed as previously described, using 400,000 cells and 1.5 ug of anti-V5 antibody or isotype control (Antibodies-online ABIN101961) per immunoprecipitation. CUT&RUN libraries were amplified with 14 cycles of PCR.

Human CD34+ hematopoietic progenitor cells were transduced with N-terminally tagged V5-*PML-RARA*^{WT}, or empty MSCV-IRES-GFP retroviruses. Cells were cultured for 6 to 8 weeks in human hematopoietic progenitor cell media until V5-*PML-RARA*^{WT} transduced GFP+ cells (but not empty vector transduced GFP+) grew out to a population frequency of 90%+ as determined by flow cytometry. Cells were then treated with 100 nM ATRA or vehicle control (DMSO) for 48 hours. The MAGnify ChIP kit (Invitrogen) was used to perform ChIP-seq. 4 million cells and 5 ug of anti-V5 antibody (Abcam) were used per immunoprecipitation. Fixed cells were resuspended in MAGnify lysis buffer at 100 million cells/mL and DNA was sheered to an average of 200 bp with a Covaris S220 (12 minutes, peak power 70, 5% duty factor, 200 cycles/burst). Lysates were diluted with 4 volumes of MAGnify dilution buffer prior to immunoprecipitation. Input DNA was isolated and sequenced from sonicated lysates as a negative control. Mouse and human ChIP-seq libraries were prepared with NEBNext Ultra II DNA Library Prep Kit (New England Biolabs).

ATAC-seq

Briefly, promyelocytes were purified as previously described (5). In separate experiments, mouse lineage-depleted bone marrow or human CD34+ hematopoietic progenitors were transduced with *V5-PML-RARA*^{WT}, *V5-PML-RARA*^{C88A}, or empty MSCV-IRES-GFP retroviruses. Seven days following the first transduction, GFP+ cells were purified by flow cytometry. Nuclei were isolated by resuspension and incubation in ice cold nuclei lysis buffer (0.1% Igepal, 10 mM Tris-HCl pH 7.4, 10 mM NaCl, 3 mM $MgCl_2$) on ice for 10 minutes. Nuclei were spun at 500 g for 10 minutes at 4°C, and then resuspended in 25 uL of Tagment TDE1 buffer (Illumina), 22.5 uL of nuclease free water, and 2.5 uL of Tagment TDE1 enzyme (Illumina). The tagmentation reaction was incubated at 37°C for 30 minutes. DNA was purified using the MinElute PCR

purification kit (Qiagen) and eluted in 10 uL of buffer EB (Qiagen). 100,000 cells were used for each replicate.

Bulk RNA sequencing, processing, and analysis

Bulk RNA-seq and analysis was performed as previously described (3).

Single-cell RNA library construction and sequencing

Single cell RNA-sequencing was performed and analyzed exactly as previously described (6). Briefly, mouse lineage-depleted bone marrow cells or human CD34+ hematopoietic progenitors were transduced with *PML-RARA*^{WT}, *PML-RARA*^{C88A}, or empty MSCV-IRES-GFP retroviruses. Seven days following the first transduction, unsorted (GFP+ plus GFP-) cells were sequenced on a 10x Genomics Chromium Controller. For *Gata2* overexpression studies, mouse lineage-depleted bone marrow cells were transduced with an MSCV-*Gata2*-IRES-mCherry vector or an empty MSCV-IRES-mCherry vector, and 4 days later mCherry+ cells were flow purified and sequenced. For *Gata2*-deficient studies, lineage-depleted bone marrow from *Ctsg-PML::RARA x Cas9-GFP* mice was transfected with CRISPR/Cas9 guide RNAs targeting *Gata2* or *Rosa26* intron 1 (3), followed by replating in MethoCult for 8 weeks (3) and sequencing; analysis was restricted to GMPs (6) for both samples and *Gata2*-deficient cells for the *Gata2*-targeted sample.

Methylcellulose colony assays

Methylcellulose assays were performed exactly as previously described, using M3434 *MethoCult* media (STEMCELL Technologies) (7). Cells were initially plated in *MethoCult* 3 days following the first transduction. For replating of CRISPR/Cas9 genome edited cells, lineage-depleted bone marrow cells from *Cas9* mice were transduced with MSCV retroviruses

containing *PML::RARA* or no insert (empty vector) 3 days following guide RNA electroporation prior to initial replating.

Western blotting and analysis

Western blots were performed and analyzed using the Jess Protein Simple western blotting system as previously described (3). Samples were blotted with mouse anti-RARA (Santa Cruz sc-515796; 1:25 dilution), rabbit anti-V5 (Abcam ab9116; 1:250), rabbit anti-Beta-Actin (Novus NB600532SS; 1:50), mouse anti-Beta-Actin (Sigma MAB 1501; 1:100 dilution), rabbit anti-GATA2 (Abcam ab109241; 1:50 dilution), HRP-conjugated anti-Rabbit (ProteinSimple), NIR-conjugated anti-mouse (Protein Simple), NIR-conjugated anti-Rabbit (ProteinSimple), and HRP-conjugated anti-mouse (Protein Simple).

CRISPR/Cas9 gene editing

Bone marrow harvested from *Ctsg-PML::RARA*$^{+/-}$ *x Rosa26-Cas9-GFP*$^{+/-}$ *(Ctsg-PML::RARA x Cas9)* mice or WT *Rosa26-Cas9-GFP*$^{+/-}$ (*Cas9*) mice was electroporated with CRISPR/Cas9 guide RNAs as previously described (3). Briefly, two guide RNAs per target (*Gata2*: one targeting upstream of the translational start codon and the other targeting downstream of the translational stop codon; *Rosa26*: one targeting upstream in intron 1 and the other targeting downstream in intron 1) were co-transfected into lineage-depleted bone marrow cells leading to deletion of the intervening sequence between the guide RNAs in greater than 50% of the targeted alleles. For each target, the frequency of targeted alleles that had insertion/deletion mutations (indels) was measured using one PCR reaction containing three primers capable of amplifying both the WT and mutated alleles. PCR products were sequenced with digital sequencing as previously described (3). Guide RNA and primer sequences can be found in SI Appendix, Table S2.

Digital sequencing and analysis of CRISPR/Cas9 targets

Digital sequencing and analysis of CRISPR/Cas9 targets exactly as previously described (3) using target specific forward and reverse primers listed in SI Appendix, Table S2.

ChIP-seq and CUT&RUN analyses

PML::RARA^{WT} binding sites in mouse cells were defined as follows. PML::RARA^{WT}-V5 ChIP-seq sites were defined compared to a DNA input control using a signal value ≥ 5 by MACS2.0 software (8). Then 150 bp were added up and downstream of each peak summit, followed by removal of duplicate and overlapping peaks (termed "PML::RARA^{WT}-V5 background subtracted peaks"). Peaks were similarly called for anti-V5 ChIP-seq in empty vector transduced cells compared to a DNA input control (termed "empty vector background subtracted peaks"). Then DiffBind (9) was used to apply Irreproducible Discovery Rate (IDR) analysis (10) to identify consensus peaks between replicates. Diffbind was then used to call differential peaks that showed a fold-change ≥ 2 and FDR ≤ 0.01 between the PML::RARA^{WT}-V5 background subtracted peaks and the empty vector background subtracted peaks using DESeq2 (termed "PML::RARA^{WT}-V5 ChIP-seq peaks"). A similar approach was taken to call PML::RARA^{WT}-V5 CUT&RUN peaks, except that an isotype control was used as a reference instead of a DNA input control. Finally, PML::RARA^{WT} binding sites were defined as those with at least a 1 bp overlap in binding between the PML::RARA^{WT}-V5 ChIP-seq peaks and the PML::RARA^{WT}-V5 CUT&RUN peaks. PML::RARA^{WT}-V5 ChIP-seq binding sites in human CD34-enriched cord bloods cells were similarly identified using MACS2.0 software by comparing differences between those peaks identified following anti-V5 ChIP-seq in cells transduced with PML::RARA^{WT}-V5 compared to a DNA input control. Density plots of relative gene expression values at loci with or without PML::RARA^{WT} binding sites within 1 kb were generated using BETA analysis (11); P value between groups determined by Kolmogorov-Smirnov test.

ATAC-seq analysis

ATAC-seq peaks were defined using a signal value ≥ 5 by MACS2.0 software (8). Then 150 bp were added up and downstream of each peak summit, followed by removal of duplicate and overlapping peaks. Then DiffBind (9) was used to apply Irreproducible Discovery Rate (IDR) analysis (10) to identify consensus peaks between replicates. Diffbind was then used to call differential peaks between experimental conditions using a threshold of FDR ≤ 0.05 and fold-change ≥ 1.5 by DESeq2. P-values for enrichment of ATAC-seq peaks within ChIP-seq or other ATAC-seq data was determined using a permutation test within the regioneR package using 10,000 permutations (12).

Peak distribution plots

Genomic distribution plots of ChIP-seq and ATAC-seq peaks was annotated as previously described (13). P-values for enrichment of datasets within specific regions was determined using a permutation test within the regioneR package using 10,000 permutations (12).

Motif enrichment

Motif enrichment plots were generated using the default settings of the known motif enrichment analysis in HOMER software v4.11 (14).

Mouse and human gene name conversion

Gene names were converted between species using the Mouse Genome Database (15). This conversion was used to compare overlapping gene lists between human and mouse datasets, and for pathway analysis.

Pathway analysis

Human gene names were used to run pathway analysis in ToppFun using the default settings (16). The Bonferroni method was used for multiple testing correction.

Immunofluorescence

150,000 cells were spun at 350 rpm for 7 minutes onto a glass slide within a hydrophobic ring drawn by a PAP pen (Thomas Scientific) onto a glass slide. Cells were fixed onto the slide with 4% paraformaldehyde (ThermoFisher R37814) for 10 minutes at room temperatures. Slide was washed for 3 times with ice-cold phosphate buffered saline (PBS) (Gibco), incubating for 5 minutes at room temperature for each wash. Cells were permeabilized and pre-blocked in IF Staining Solution (PBS containing 0.1% saponin [Sigma], 1% bovine serum albumin [Sigma]), and 10% normal goat serum (which matches secondary antibody host) for 30 to 120 minutes at 4°C. Slide was washed 5 times with PBS, and then incubated with primary antibody (1:1000 dilution of anti-BirA [TurboID] Novus NBP2-59939; or 1:50 dilution of anti-PML Santa Cruz sc-966) in IF Staining Solution within a humidified chamber using damp tissue paper (Kimberly Clark). Slide was washed 5 times with PBS, and then incubated with secondary antibody (1:500 dilution of goat anti-Mouse Alexa Fluor 633 (ThermoFisher A-21051) and 1 ug/mL DAPI (ThermoFisher 62248) for 45-120 minutes in IF Staining Solution at room temperature in the dark. Slide was washed 5 times with PBS, then mounted with ProLong Glass Antifade (ThermoFisher P36982), and cured overnight at 4°C in the dark. Slides were imaged with an Echo Revolve microscope.

Proximity labeling and mass spectrometry

Lineage-depleted bone marrow cells from C57Bl/6 mice were transduced with MSCV-IRES-GFP retroviruses containing *TurboID-PML::RARA*^{WT}*, TurboID-PML::RARA*^{C88A}*, PML::RARA*^{WT}-

TurboID, or a *TurboID* cDNA alone. Proximity labeling and mass spectrometry were performed as previously described (17).

Ultra high performance liquid chromatography mass spectrometry

Peptides were prepared using an on-bead tryptic digestion as previously described (17), and analyzed using trapped ion mobility time-of-flight mass spectrometry as previously described (17). Briefly, peptides were separated using a *nano-ELUTE*® chromatograph (Bruker Daltonics. Bremen, Germany) interfaced to a timsTOF Pro 2 mass spectrometer (Bruker Daltonics) with a modified nano-electrospray source (CaptiveSpray, Bruker Daltonics).

Proximity labeling and mass spectrometry analysis

Peptides were mapped to mouse proteins as previously described (17). Spectral counts were normalized to spectral counts per million (CPM) for each sample, as previously described (17). Spectral counts of detected proteins were compared against controls using the edgeR (18), and differentially interacting proteins were defined as having an FDR ≤ 0.05 and fold-change ≥ 2. A minimum of 6 spectral counts were required in at least 50% of the samples in the numerator group for a protein to be considered detected above background. For example, at least 6 spectral counts were required in at least 50% of the PML::RARA^{WT}-TurboID and TurboID-PML::RARA^{WT} samples (numerator group) when calling differentially interacting proteins between PML::RARA^{WT} and TurboID cDNA alone (denominator group). PML::RARA^{WT} interacting proteins were called using one comparison of the PML::RARA^{WT}-TurboID and TurboID-PML::RARA^{WT} samples compared to the TurboID cDNA alone samples.

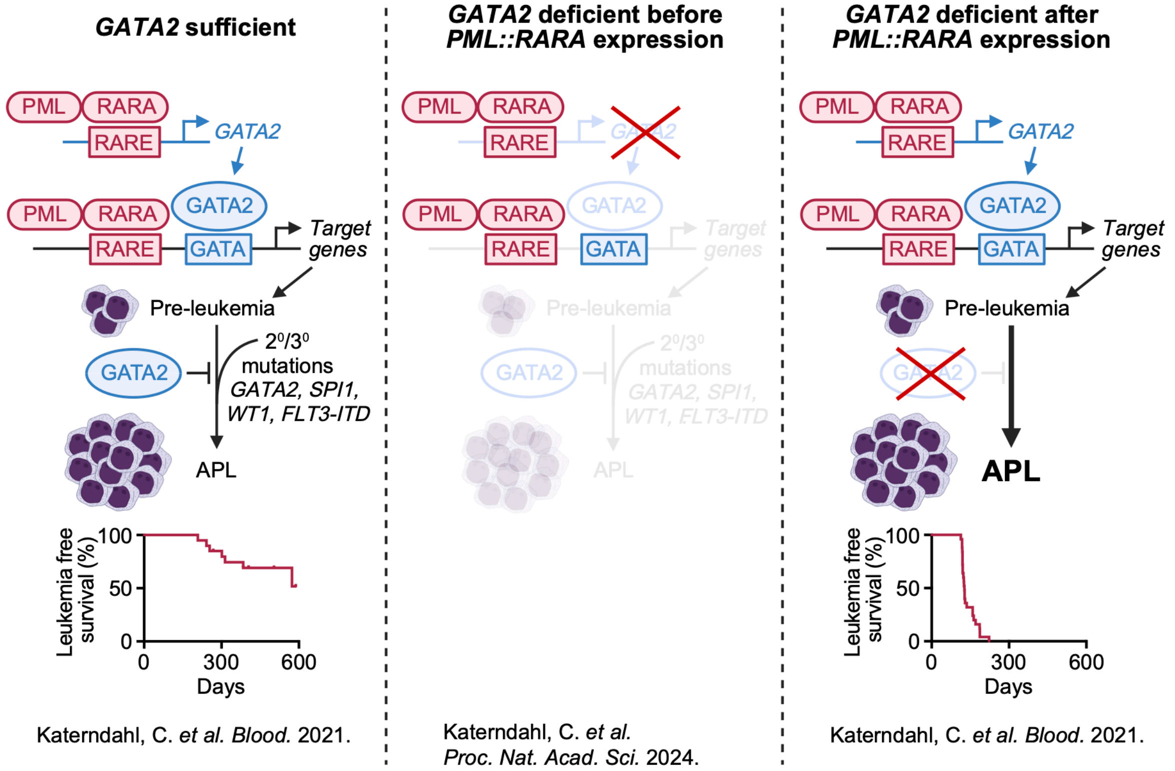

Fig. S1. Model of PML::RARA and GATA2 cooperation to initiate acute promyelocytic leukemia. Left panel: In *GATA2*-sufficient cells, PML::RARA binds to the DNA sequences near the *GATA2* locus and induces *GATA2* expression. GATA2 and PML::RARA then coordinately regulate target genes by binding to closely spaced GATA and RARE motifs in the genome. The pre-leukemic transcriptional phenotype induced by PML::RARA is dependent upon *GATA2* . However, once the pre-leukemic program is established, *GATA2* is dispensable, and in fact, acts as a tumor suppressor to limit proliferative stress. Signaling mutations (e.g. *FLT3*-ITD), or inactivating mutations in tumor suppressors (e.g. *GATA2, SPI1,* or *WT1*) can cooperate with *PML::RARA* to promote the development of APL. Middle panel: In cells that are *GATA2*-deficient prior to *PML::RARA* expression, *PML::RARA* is unable to induce its pre-leukemic reprogramming phenotype. Right panel: *PML::RARA* reprograms cells that are initially *GATA2*-sufficient. Subsequent loss of *GATA2* leads to highly penetrant APL with short latency. This model may explain why loss-of-function *GATA2* mutations (and/or epigenetic silencing of *GATA2*) can cooperate with *PML::RARA* (and other AML-initiating mutations) to promote the development of AML. Kaplan-Meyer plots at the bottom of the left and right panels are modified from the data presented in Katerndahl *et al.* (3).

A

Publication	Technique	Target	Direct / indirect identification of binding sites	Cell type	Replicates	Negative controls
Katerndahl *et al.*	ChIP-seq	V5-PML::RARA	Direct	Primary lineage-depleted mouse bone marrow	2	Empty vector, input DNA
	CUT&RUN	V5-PML::RARA	Direct	Primary lineage-depleted mouse bone marrow	3	V5-PML::RARA^{C88A}, empty vector, isotype
	ChIP-seq	V5-PML::RARA	Direct	Primary human CD34 enriched cord blood	3	Input DNA, +/-ATRA
Wang *et al.*	ChIP-on-chip	PML & RARA	Indirect (inferred bioinformatically)	PR9 cell line	3	Input DNA
Martens *et al.*	ChIP-seq	PML & RARA	Indirect (inferred bioinformatically)	NB4 cell line	1	Input DNA
	ChIP-seq	PML & RARA	Indirect (inferred bioinformatically)	PR9 cell line	1	Input DNA, +/-Zinc
	ChIP-seq	PML	Direct	Primary APL	2	None
Tan *et al.*	ChIP-seq	PML::RARA fusion site	Direct	NB4 cell line	1	Input DNA

B

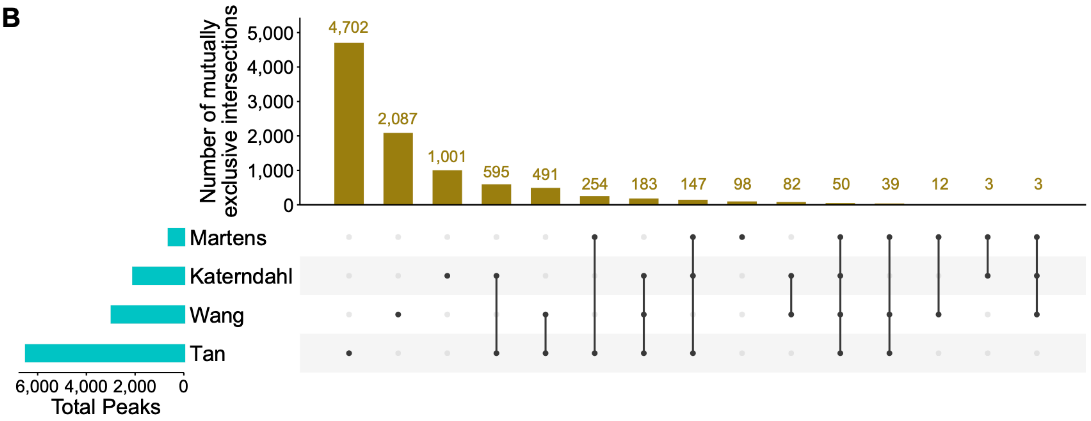

C

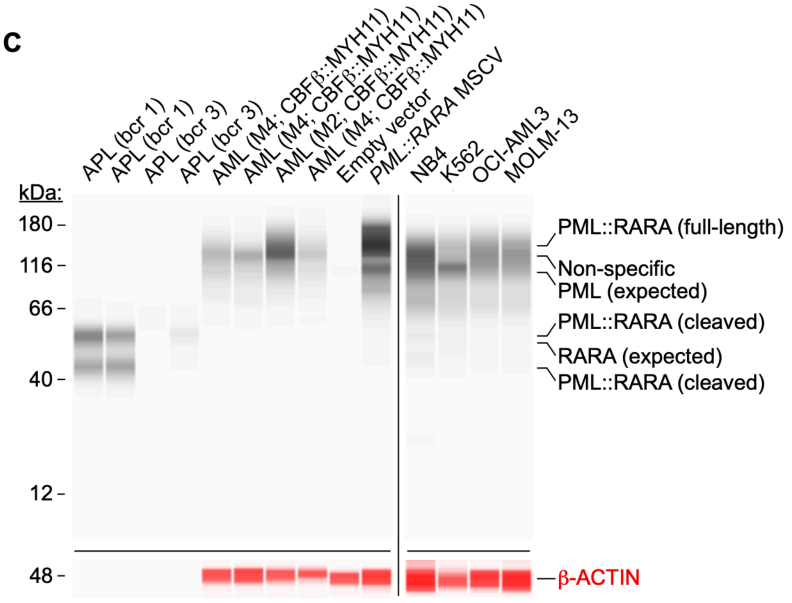

Fig. S2. Previous studies of PML::RARA binding sites in the genome. (A) Summary of studies to date that have examined the genomic binding sites of PML::RARA. These studies vary in the techniques, target protein, cell types, replicates, controls, and whether binding sites were detected directly or indirectly; Wang *et al.* (19) and Martens *et al.* (20) used antibodies specific to native PML and RARA, and then then bioinformatically inferred PML::RARA binding at sites that

showed overlap for the binding of both proteins. In contrast, Tan *et al.* (21) used a commercially available antibody raised against a 200 amino acid long peptide containing the PML::RARA fusion (see panel C). (B) "Upset plot" illustrating the number of mutually exclusive intersections between the PML::RARA binding sites identified in each study (vertical dark yellow bars) (19-21). Horizontal light blue bars indicate the total number of binding sites identified in each study. (C) Western blot analysis using a commercially available antibody (ABclonal #: A7525) raised against a 200 amino acid long peptide containing the fusion site of the bcr1 isoform of PML::RARA (21). Samples are from 2 independent primary human APL bone marrow samples with the bcr1 isoform, 2 independent APL bone marrow samples with the bcr3 isoform, 4 independent AML cases with *CBFB::MYH11* fusions (3 with M4 subtype and 1 with M2 subtype), lineage-depleted mouse bone marrow cells transduced with MSCV based retroviruses containing a bcr1 isoform of human *PML::RARA* cDNA or no insert (empty vector), and NB-4 cells (a cell line derived from an APL sample with the bcr1 isoform), K562 cells, OCI-AML3 cells, or MOLM-13 cells. Note the lack of Beta-ACTIN, and the cleavage of PML::RARA in the primary APL cases, most likely due to the cleavage of both proteins by the abundant serine protease ELANE in these samples (22). This antibody also recognizes one or more protein(s) in samples that do not express PML::RARA (non-M3 AMLs, K562, MOLM-13, and OCI-AML3 cells).

A

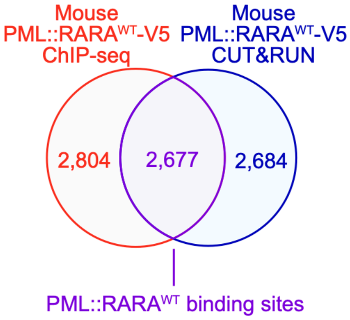

Fig. S3. Overlap of the binding sites of V5-tagged PML::RARA identified by ChIP-seq and CUT&RUN. (A) Venn diagram illustrating the sequence-level overlap (minimum of 1 bp) of the mouse PML::RARA-V5 ChIP-seq and CUT&RUN peaks. PML::RARA^{WT} binding sites were defined as those regions (2,677) with PML::RARA binding by both ChIP-seq and CUT&RUN.

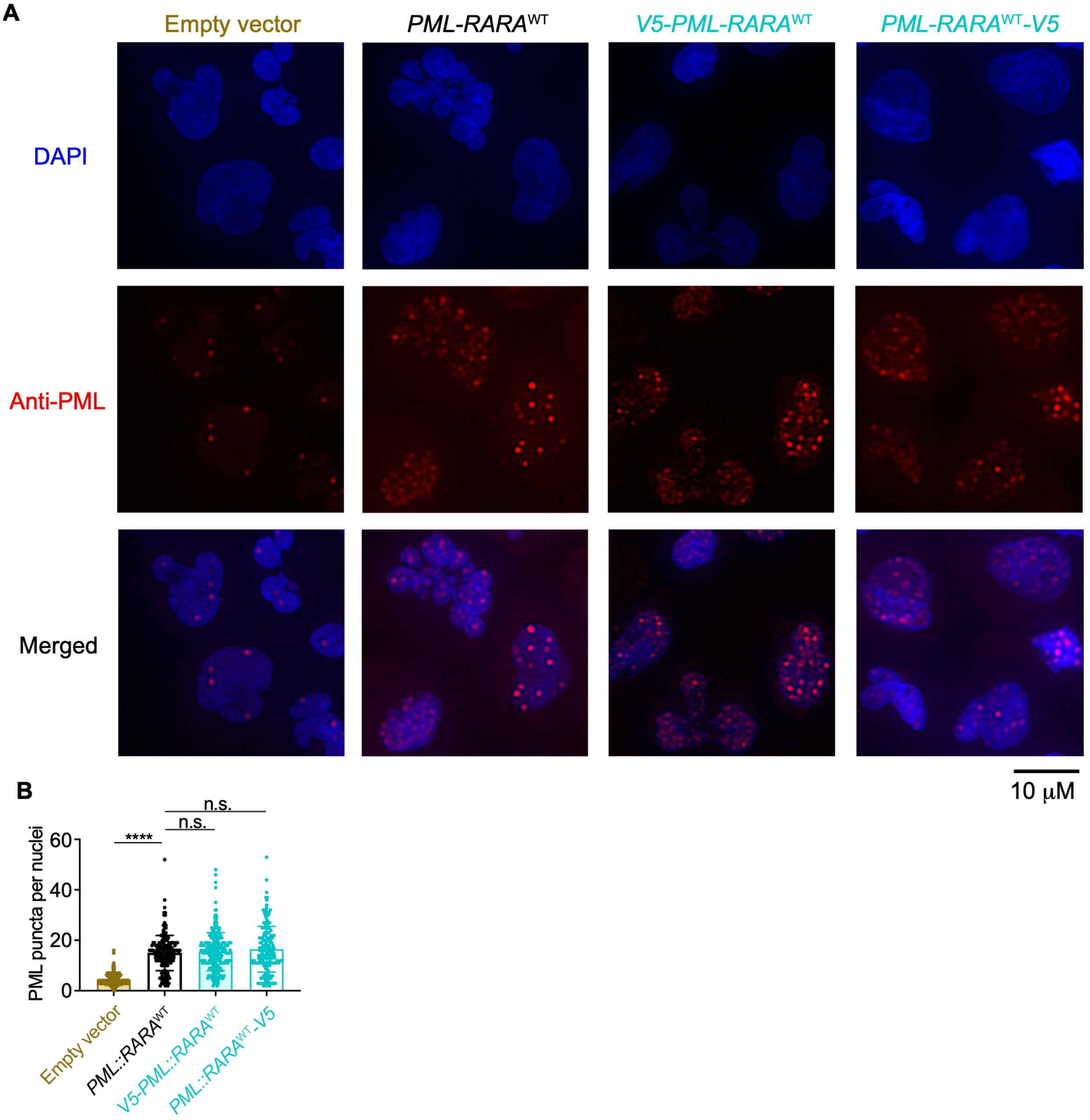

Fig. S4. V5 tagging does not interfere with the ability of PML::RARA to reorganize PML nuclear bodies into microspeckles. (A). Immunofluorescence of CD34 enriched human cord blood cells transduced with MSCV-IRES-GFP based retroviruses containing *PML::RARA*^{WT}, *V5-PML::RARA*^{WT}, *PML::RARA*^{WT}*-V5*, or an empty vector. GFP+ cells were flow purified and stained with DAPI (blue) and an anti-PML antibody (red). Images are from a sample that is representative of three independent biological replicates. (B) Quantification of the number of PML puncta per nuclei in the cells from (A). Data are from three independent biological replicates. ****$P < 0.0001$, n.s. = not significant by two-way ANOVA.

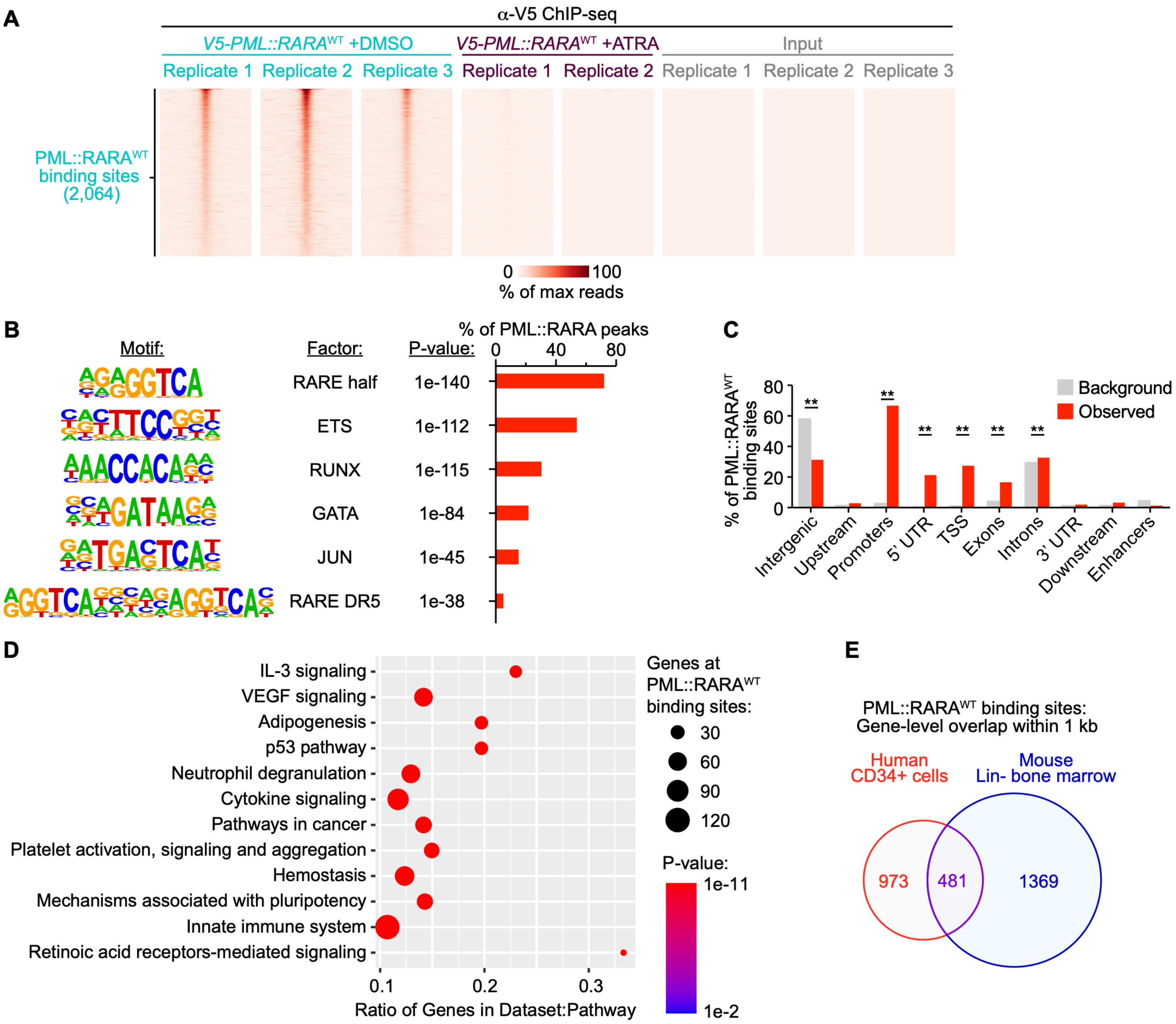

Fig. S5. Identification of the binding sites of V5-tagged PML::RARA in primary human hematopoietic progenitor cells by ChIP-seq. (A) "Tornado plots" of anti-V5 ChIP-seq in cells transduced with *V5-PML::RARA*^{WT} and treated with 100 nM ATRA or DMSO (vehicle control) for 48 hours. PML::RARA^{WT} binding sites (regions bound by V5-PML::RARA^{WT} in cells treated with DMSO) are plotted along the Y-axis, ordered from top to bottom in greatest to least V5-PML::RARA^{WT} enrichment. Each replicate panel represents an independent biological replicate. (B) Motif enrichment at PML::RARA^{WT} binding sites by HOMER analysis (13). (C) Distribution of PML::RARA^{WT} binding sites at various regions in the genome compared to that of the mm10 reference genome (background). **P < 0.01. (D) Pathway enrichment at PML::RARA^{WT} binding sites. Genes within 1 kb of binding sites were analyzed. (E) Venn diagram illustrating the gene-level overlap between genes within 1 kb of PML::RARA^{WT} genomic binding sites identified in mouse vs. human hematopoietic cells.

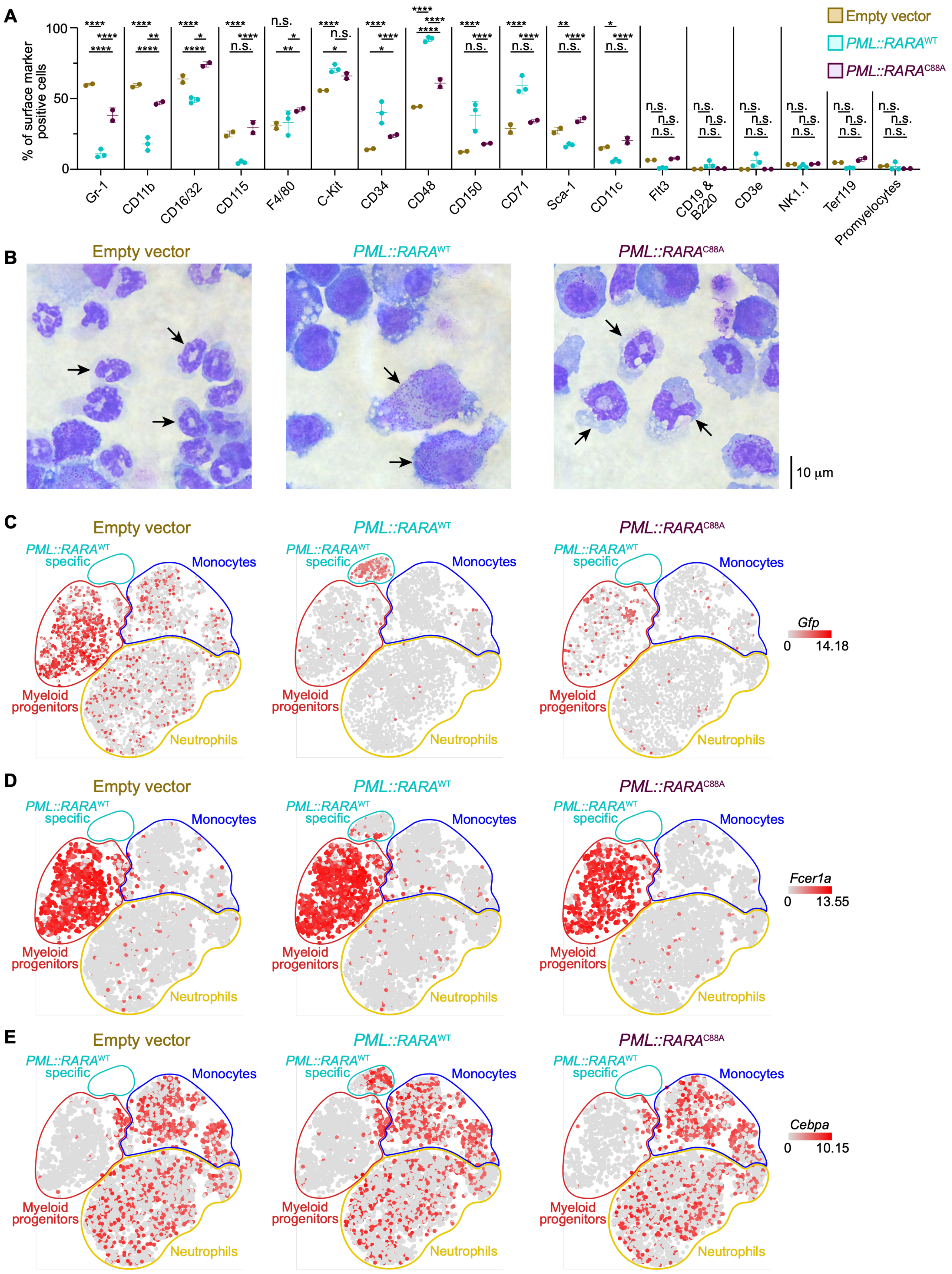

A
Empty vector
PML::RARA^WT
PML::RARA^C88A
% of surface marker positive cells
100
50
0
Gr-1
CD11b
CD16/32
CD115
F4/80
C-Kit
CD34
CD48
CD150
CD71
Sca-1
CD11c
Flt3
CD19 & B220
CD3e
NK1.1
Ter119
Promyelocytes
B
Empty vector
PML::RARA^WT
PML::RARA^C88A
10 μm
C
Empty vector
PML::RARA^WT
PML::RARA^C88A
PML::RARA^WT specific
Monocytes
Myeloid progenitors
Neutrophils
Gfp
0
14.18
D
Fcer1a
0
13.55
E
Cebpa
0
10.15

Fig. S6. *PML::RARA* expression leads to a unique population of immature myeloid cells. Phenotypic analyses of bone marrow from 8- to 12-week-old WT mice transduced with MSCV-IRES-GFP based retroviruses containing no insert ("empty vector"), *PML::RARA*^{WT}, or *PML::RARA*^{C88A}, and evaluated 7 days later. (A). Summary data of the percentage of GFP+ transduced cells that express the following surface markers by flow cytometry: Gr-1, CD11b, CD16/32, CD115, F4/80, C-Kit, CD34, CD48, CD150, CD71, Sca-1, CD11c, Flt3, CD19, and B220 simultaneously, CD3e, NK1.1, and Ter119. Also shown are the percentage of GFP+ transduced cells that are promyelocytes (B220-, CD3e-, NK1.1-, Ter119-, CD115-, Gr-1^{int}, and SSC-A$^{lo\text{-}int}$). Note that the proportion of cells that express mature myeloid surface markers (such as Gr-1, CD11b, and CD115) is lower in the cells transduced with *PML::RARA*^{WT} compared to those transduced with an empty vector or *PML::RARA*^{C88A}. Conversely, the proportion of cells that express early myeloid surface markers (such as C-Kit and CD34) is higher in the cells transduced with *PML::RARA*^{WT}. Statistical significance determined by 2-way ANOVA. *$P < 0.05$, **$P < 0.01$, ****$P < 0.0001$, n.s. = not significant. Each data point is an independent biological replicate. (B) Wright-Giemsa staining of flow-purified GFP+ cells. Note the higher frequency of mature neutrophils in the cells transduced with an empty vector or *PML::RARA*^{C88A} (highlighted with arrows) compared to those transduced with *PML::RARA*^{WT}. By contrast, the *PML::RARA*^{WT} expressing cells show early myeloid features with azurophilic granules (highlighted with arrows). Images were captured using an *Echo Revolve* at 100x original magnification. Images are representative of two independent biological replicates. (C-E) t-SNE plots of the relative expression of *Gfp* (C), *Fcer1a* (D), and *Cebpa* (E) in unsorted (GFP+ and GFP-) cells by scRNA-seq. Note that GFP+ cells transduced with *PML::RARA*^{WT} do not express the mast cell marker *Fcer1a*, but do express the myeloid-specific transcription factor *Cebpa*.

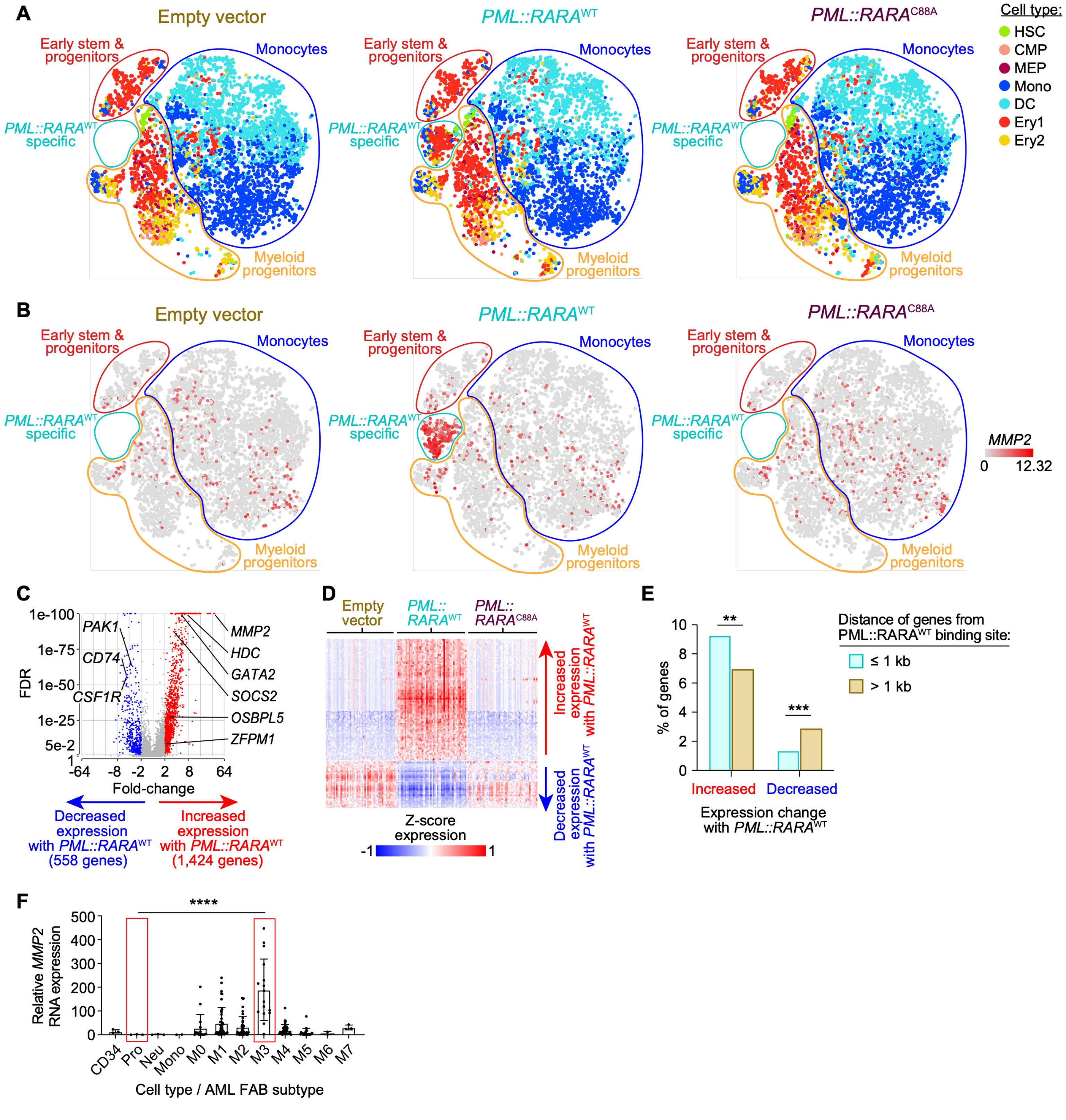

Fig. S7. Single cell RNA sequencing (scRNA-seq) following *PML::RARA* overexpression in primary human hematopoietic cells. CD34 enriched human cord blood cells were transduced with MSCV-IRES-GFP based retroviruses containing no insert ("empty vector"), *PML::RARA*^{WT}, or *PML::RARA*^{C88A}, and evaluated by scRNA-seq 7 days later. (A) t-Distributed stochastic neighbor embedding (t-SNE) plots with lineage assignment based on Haemopedia gene expression profiling (6, 13). HSC = hematopoietic stem cell, CMP = common myeloid progenitor, MEP = megakaryocyte/erythroid progenitor, Mono = monocyte, DC = dendritic cell, Ery = erythrocyte. (B) t-SNE plots of the relative expression of *MMP2*. A unique population of myeloid precursor cells that are only present in cells transduced with *PML::RARA*^{WT} is outlined in light blue ("*PML::RARA*^{WT} specific"). (C) Volcano plot of expression changes between GFP+ *PML::RARA*^{WT} vs. empty vector transduced cells. (D) Heat map of the 1,982 differentially expressed genes (DEGs) in GFP+ *PML::RARA*^{WT} vs. empty vector transduced cells (FDR $\leq$ 0.05 and fold change $\geq$ 2). *PML::RARA*^{C88A} transduced cells are passively plotted on the right.

(E) Bar graph of the percentage of genes within 1 kb or greater than 1 kb from a PML::RARA^{WT} binding site that show an increase or decrease in GFP+ *PML::RARA*^{WT} vs. empty vector transduced cells by scRNA-seq. ***P < 0.0001, ****P < 1E-14 by Fisher's exact text. (F) *MMP2* expression in flow purified healthy donor human CD34+ progenitors (CD34), promyelocytes (Pro), neutrophils (Neu), monocytes (Mono), and the AML French-American-British subtypes M0-M7 by RNA-seq using the AML TCGA data set (23). ****FDR < 1e-7.

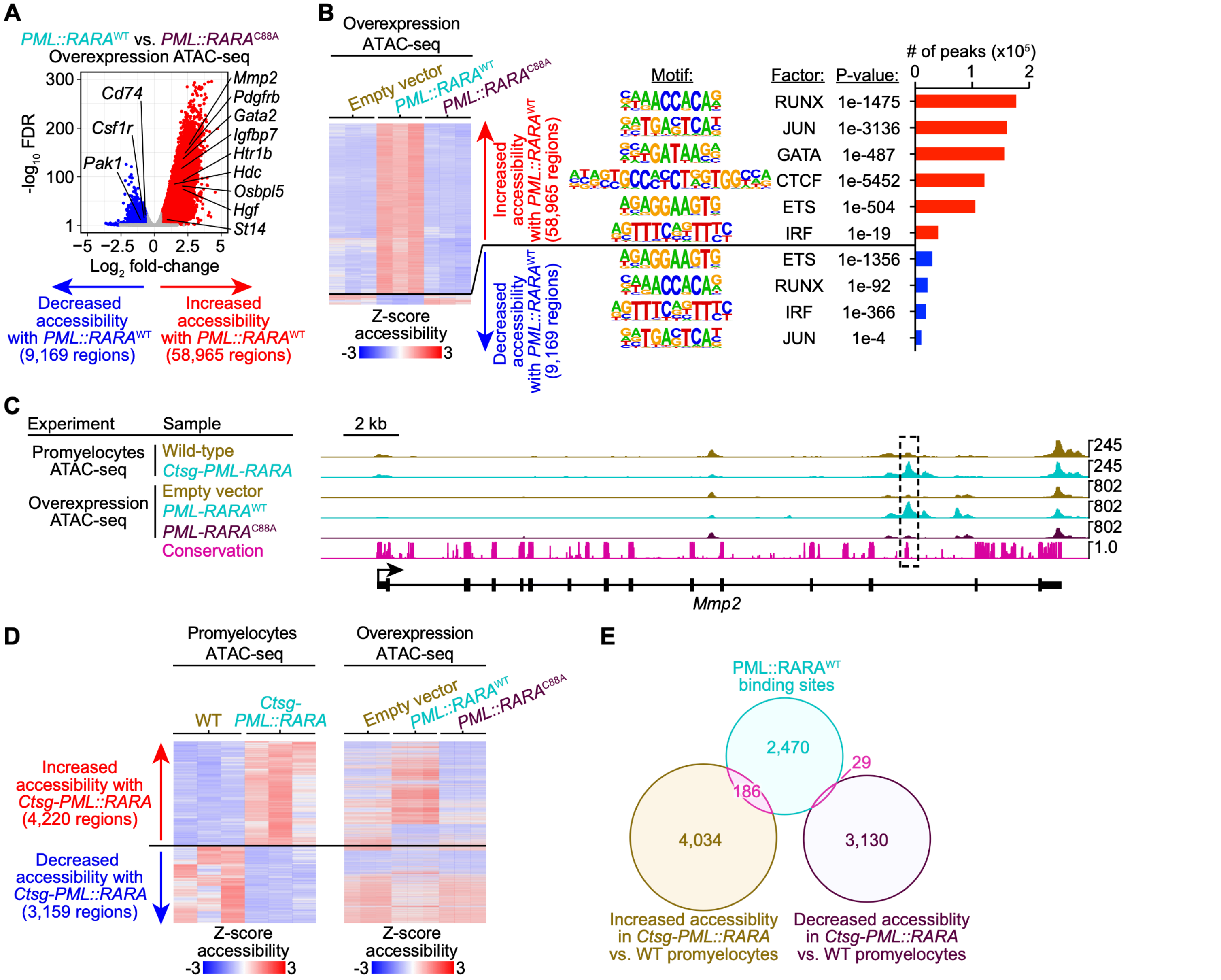

Fig. S8. *PML::RARA* overexpression vs. *Ctsg-PML::RARA* expression in bone marrow cells result in similar changes in DNA accessibility. (A) Assay for Transposase-Accessible Chromatin with sequencing (ATAC-seq) was performed on lineage-depleted mouse bone marrow cells transduced with MSCV-IRES-GFP based retroviruses containing no insert ("empty vector"), *PML::RARA*^{WT}, or *PML::RARA*^{C88A}. The cells were grown in SCF, FLT3L, IL-3, and TPO for an additional 7 days, and were then evaluated by ATAC-seq. A volcano plot of DNA accessibility changes between cells transduced with *PML::RARA*^{WT} vs. *PML::RARA*^{C88A} is shown. (B) Left panel: Heat map of the 68,134 differentially accessible regions between *PML::RARA*^{WT} vs. *PML::RARA*^{C88A} transduced cells by ATAC-seq (FDR $\leq$ 0.05 and fold change $\geq$ 1.5). Empty vector transduced cells are passively plotted. Each column represents an independent biological replicate (3 per condition). Right panel: Motif enrichment using HOMER analysis (14) at regions that show increased or decreased accessibility in *PML::RARA*^{WT} vs. *PML::RARA*^{C88A} transduced cells by ATAC-seq. (C) Genome browser tracks for the *Mmp2* locus. Conservation extent across 20 vertebrate species (24) is plotted on the bottom track. Y-axis for the ATAC-seq data represents the mean read depth per bp. The Y-axis for the conservation track is the proportion of vertebrates conserved at each region. (D) Heat maps of the 7,379 differentially accessible regions between *Ctsg-PML::RARA* vs. WT promyelocytes by ATAC-seq. Left panel: ATAC-seq data from *Ctsg-PML::RARA* vs. WT promyelocytes. Right panel: ATAC-seq from empty vector, *PML::RARA*^{WT}, or *PML::RARA*^{C88A} transduced cells are passively plotted at the *Ctsg-PML::RARA* vs. WT promyelocyte differentially accessible regions. Each column represents an independent biological replicate (3 per condition). (E) Venn diagram illustrating the sequence-level overlap (minimum of 1 bp) of the mouse PML::RARA-V5 binding sites (by ChIP-seq CUT&RUN) and the regions that showed increased or decreased accessibility in *Ctsg-PML::RARA* vs. WT promyelocytes by ATAC-seq.

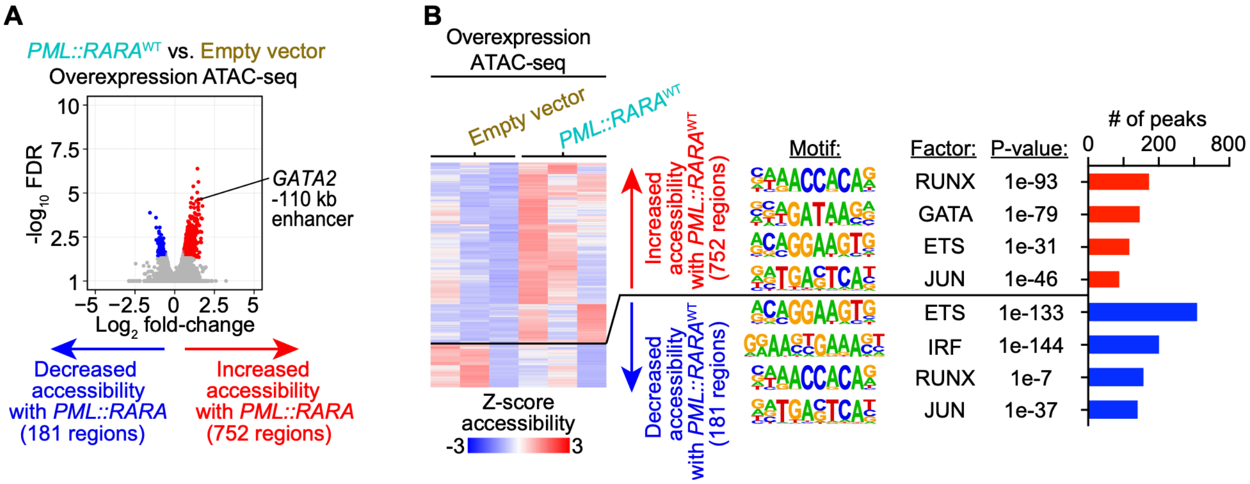

Fig. S9. GATA DNA binding motifs are enriched at regions that show increased DNA accessibility following *PML::RARA* expression in primary human hematopoietic cells. ATAC-seq was performed on CD34 enriched human cord blood cells that were transduced with MSCV-IRES-GFP based retroviruses containing no insert ("empty vector"), *PML::RARA*^{WT}, or *PML::RARA*^{C88A}, and evaluated 7 days later. (A) Volcano plot of DNA accessibility changes between cells transduced with *PML::RARA*^{WT} vs. empty vector. Accessibility changes at the *GATA2* -110 kb enhancer (25, 26) are labeled. (B) Left plot: Heat map of the 933 differentially accessible regions between *PML::RARA*^{WT} vs. empty vector transduced cells by ATAC-seq (FDR $\leq$ 0.05 and fold change $\geq$ 1.5). Each column represents an independent biological replicate (3 per condition). Right plot: Motif enrichment using HOMER analysis (14) at regions that show increased or decreased accessibility in *PML::RARA*^{WT} vs. empty vector transduced cells by ATAC-seq.

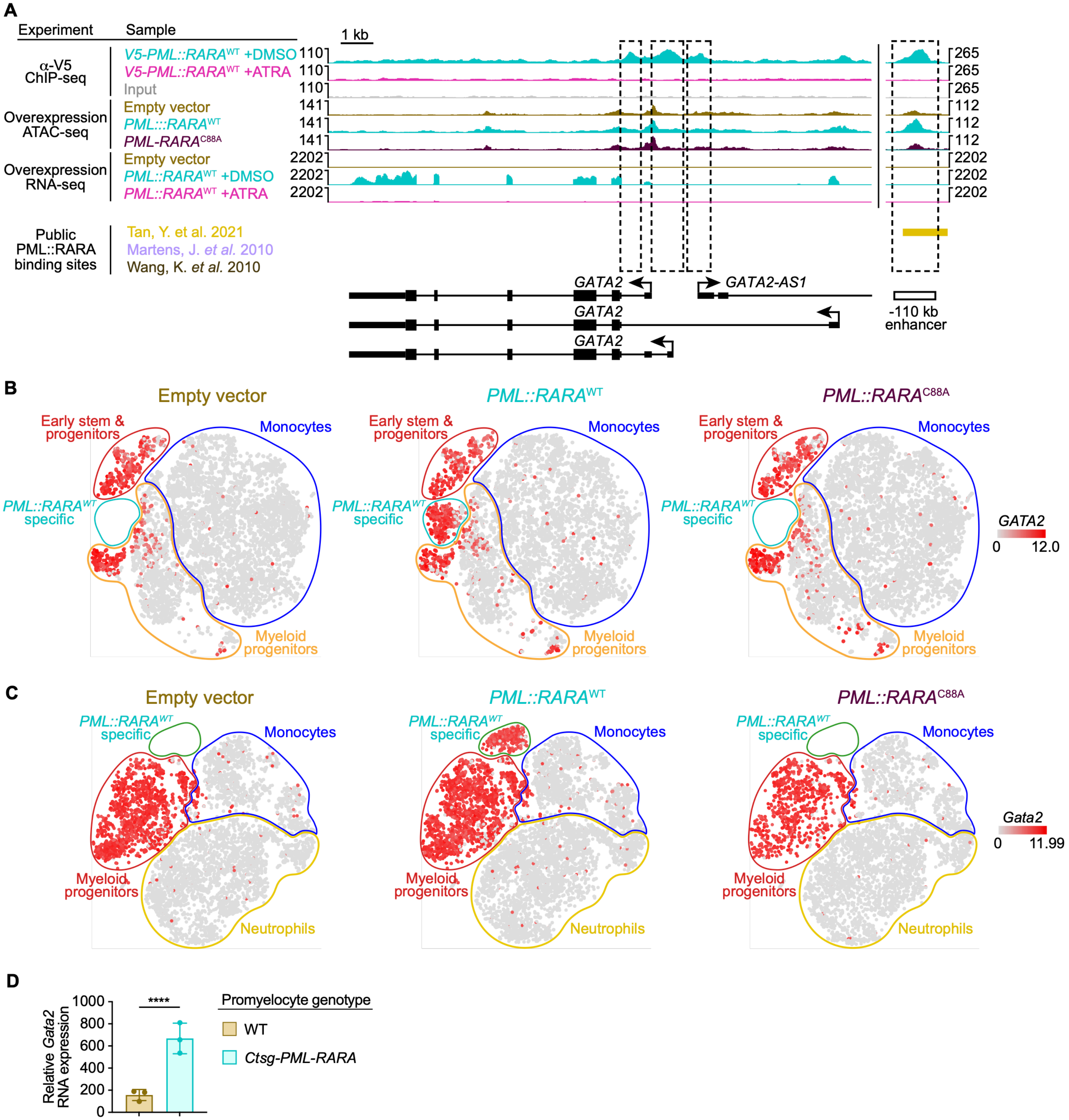

Fig. S10. PML::RARA binds to the *GATA2* locus, and leads to increases in DNA accessibility and expression in primary hematopoietic cells. (A) Genome browser tracks for the human *GATA2* gene body and -110 kb enhancer (25, 26). Dashed boxes highlight regions with PML::RARA^{WT} binding sites. Note that the *GATA2* -110 kb enhancer is bound by PML::RARA^{WT}, and shows increased DNA accessibility with *PML::RARA*^{WT} expression. Any PML::RARA binding sites identified in previous PML::RARA genomic binding sites studies are plotted in the bottom three tracks; note that Tan, Y. *et al.* (21) detected PML::RARA binding at the *GATA2* enhancer, but not in the *GATA2* gene body. Y-axis is the mean read depth per bp. (B) t-SNE plots of the relative expression of *GATA2* in empty vector, *PML::RARA*^{WT}, or *PML::RARA*^{C88A} transduced human CD34+ cells by scRNA-seq. A unique population of myeloid precursor cells that is only present in cells transduced with *PML::RARA*^{WT} is outlined in light blue ("*PML::RARA*^{WT} specific"). (C) t-SNE plots of the relative expression

of *Gata2* in empty vector, *PML::RARA*^{WT}, or *PML::RARA*^{C88A} transduced mouse lineage depleted bone marrow cells by scRNA-seq. Note that *Gata2* was highly expressed in the GFP+ *PML::RARA*^{WT} transduced population, but was also highly expressed in GFP untransduced cells and GFP+, empty vector transduced cells. Although *Gata2* did not meet the 2-fold change threshold required to be a differentially expressed gene, it was significantly upregulated. (D) Relative *Gata2* RNA expression in flow-purified promyelocytes from pre-leukemic *Ctsg-PML-RARA* mice (compared to those from WT mice by RNA-seq. P-values presented are corrected for multiple testing. ****P < 0.0001.

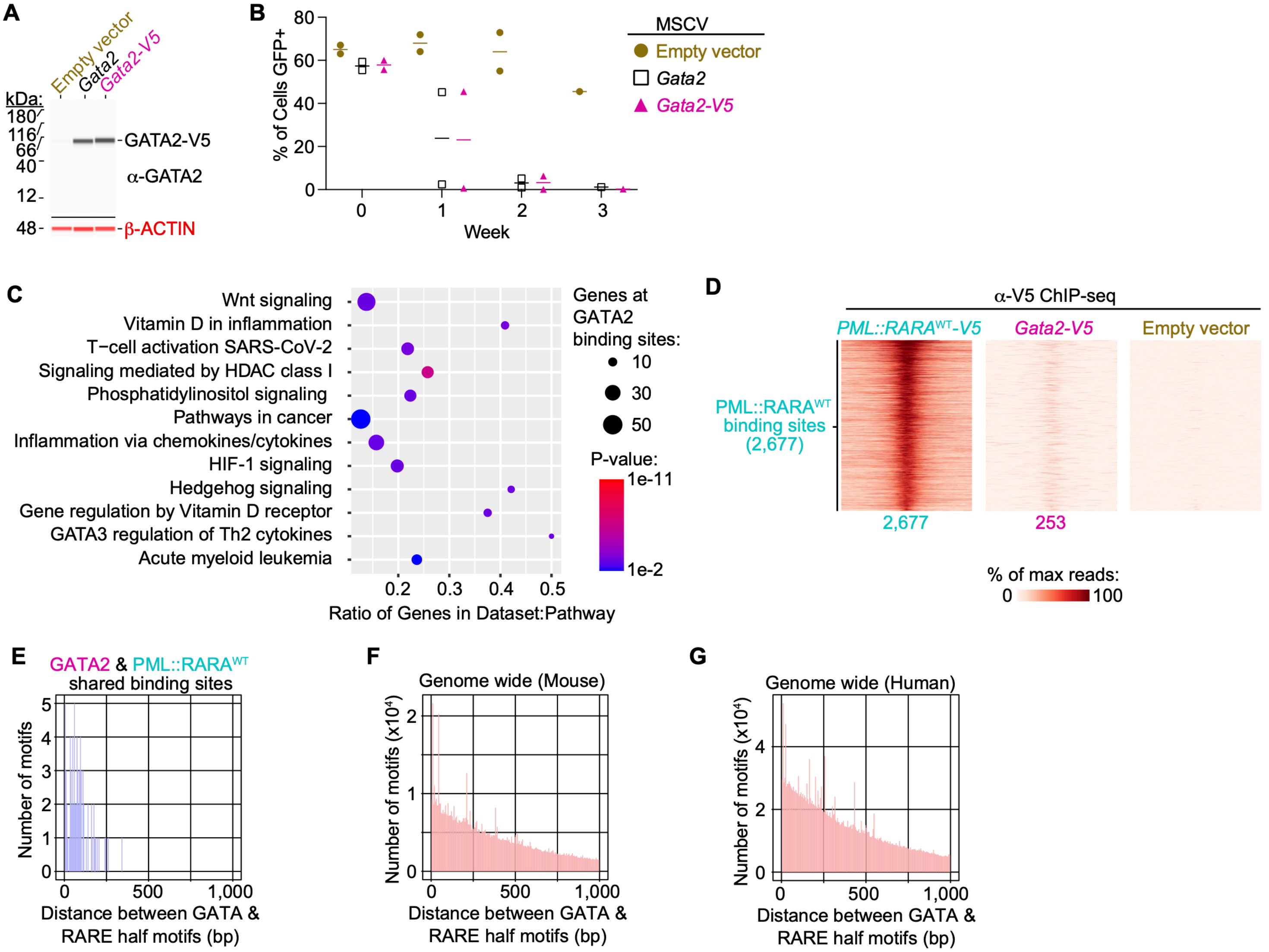

Fig. S11. GATA2 binds near PML::RARA^{WT} binding sites by utilizing a network of closely spaced RARE and GATA motifs in the genome. (A) Anti-GATA2 (top blot) or anti-beta actin (bottom blot) western blot analysis in cells transduced with *Gata2*, *Gata2-V5* or an empty vector, four days following transduction. Each lane is from an independent sample, representative of two replicates. (B) Colony counts from serial replating assays in which transduced cells from A were replated in *Methocult*® M3434. Two independent biological replicates are plotted for each condition. (C) Pathway enrichment at GATA2 binding sites. Genes within 1 kb of binding sites were analyzed. (D) Tornado plots of the 2,677 PML::RARA^{WT} binding sites plotted along the Y-axis. Anti-V5 ChIP-seq in cells retrovirally transduced with *Gata2-V5* or an empty vector are passively plotted at the PML::RARA^{WT} binding sites. 253 of the 2,677 PML::RARA^{WT} binding sites are also bound by GATA2. (E-F) Relative distance in bp between retinoic acid response element (RARE) half motifs (RGGTCA) and GATA motifs (WGATAR) at the 253 regions bound by GATA2 and PML::RARA^{WT} (E), and in the mouse (F) and human genomes (G).

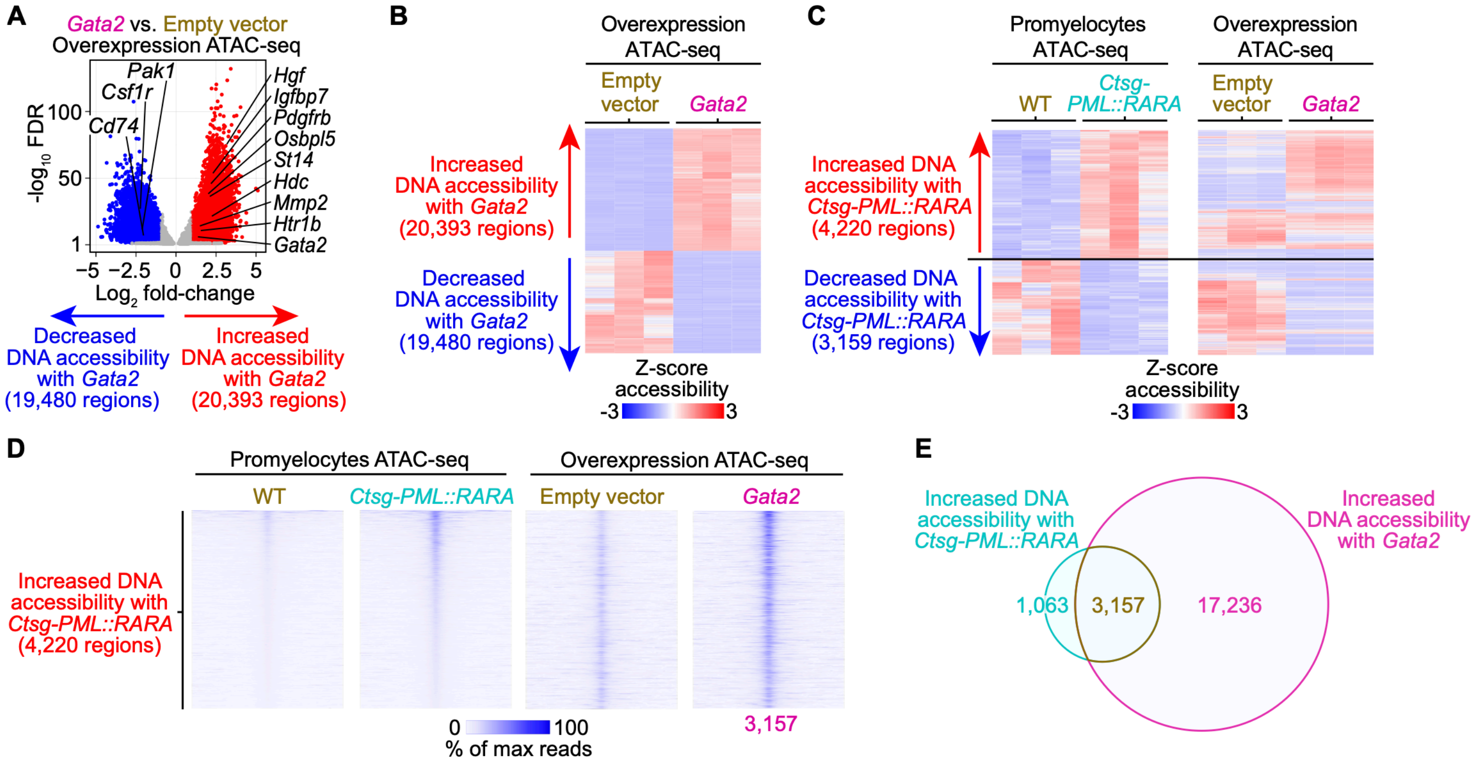

Fig. S12. Expression of *Gata2* and *PML::RARA* lead to similar changes in DNA accessibility. (A) ATAC-seq was performed on lineage-depleted mouse bone marrow cells that were transduced with MSCV-IRES-GFP based retroviruses containing no insert ("empty vector") or *Gata2*, and evaluated 4 days later. Volcano plot of DNA accessibility changes between cells transduced with *Gata2* vs. empty vector. (B) Heat map of the 39,873 differentially accessible regions between *Gata2* vs. empty vector transduced cells by ATAC-seq (FDR $\geq$ 0.05 and fold change $\leq$ 1.5). Each column represents an independent biological replicate (3 per condition). (C) Heat maps of the 7,379 differentially accessible regions between *Ctsg-PML::RARA* vs. WT promyelocytes by ATAC-seq. Left plot: ATAC-seq data from *Ctsg-PML::RARA* vs. WT promyelocytes. Right plot: ATAC-seq from empty vector or *Gata2* transduced cells are passively plotted at the *Ctsg-PML::RARA* vs. WT promyelocytes differentially accessible regions. Each column represents an independent biological replicate (3 per condition). (D) Tornado plots of the 4,220 regions that showed increased DNA accessibility in *Ctsg-PML::RARA* vs. WT promyelocytes by ATAC-seq plotted along the Y-axis. ATAC-seq in cells retrovirally transduced with *Gata2* or an empty vector are passively plotted. 3,157 of the 4,220 regions that showed increased DNA accessibility in *Ctsg-PML::RARA* vs. WT promyelocytes also showed increased accessibility in *Gata2* vs. empty vector transduced cells. (E) Venn diagram illustrating the sequence-level overlap (minimum of 1 bp) between the regions that showed increased DNA accessibility with *Ctsg-PML::RARA*, and those that showed increased DNA accessibility with *Gata2* expression.

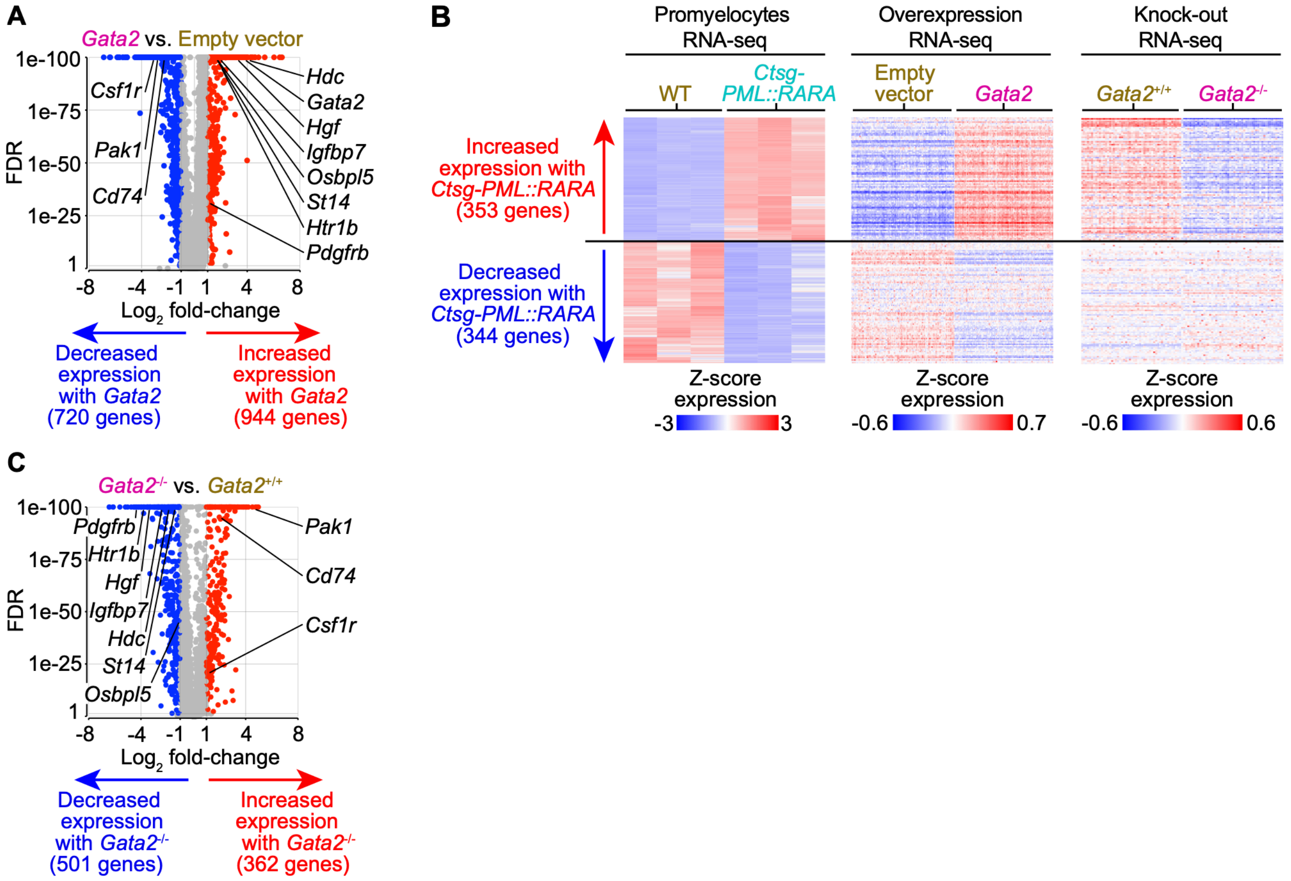

Fig. S13. Expression of *Gata2* and *PML::RARA* lead to similar changes in RNA expression. (A) Single cell RNA sequencing (scRNA-seq) of lineage-depleted WT mouse bone marrow cells that were transduced with MSCV-IRES-mCherry based retroviruses containing no insert ("empty vector") or *Gata2* cDNA. mCherry+ cells were flow purified and evaluated 4 days later. Volcano plot of expression changes between *Gata2* vs. empty vector transduced cells. (B) Heat maps of the 697 DEGs between *Ctsg-PML::RARA* vs. WT promyelocytes by RNA-seq (FDR $\leq$ 0.05 and fold change $\geq$ 2). Left panel: Bulk RNA-seq of *Ctsg-PML::RARA* or WT promyelocytes. Each column represents an independent biological replicate (3 per genotype). Center panel: scRNA-seq of cells transduced with *Gata2* vs. empty vector, passively plotted at the 697 DEGs from the left plot. Each column represents an individual cell. Analysis was restricted to granulocyte-monocyte progenitors (GMPs) using lineage assignment software (6, 13) to minimize expression changes that may be due to differentiation states. Right panel: Passively plotted scRNA-seq from lineage-depleted *Ctsg-PML::RARA* x *Cas9-GFP* mouse bone marrow cells that were targeted with CRISPR/Cas9 guide RNAs specific to *Gata2* (*Gata2*$^{-/-}$) or *Rosa26* intron 1 (*Gata2*$^{+/+}$; neutral mutation control) and replated in *MethoCult* M3434 for 8 weeks and then evaluated. Each column represents a single cell. (C) Volcano plot of expression changes between *Gata2*$^{-/-}$ vs. *Gata2*$^{+/+}$ GMPs from (B).

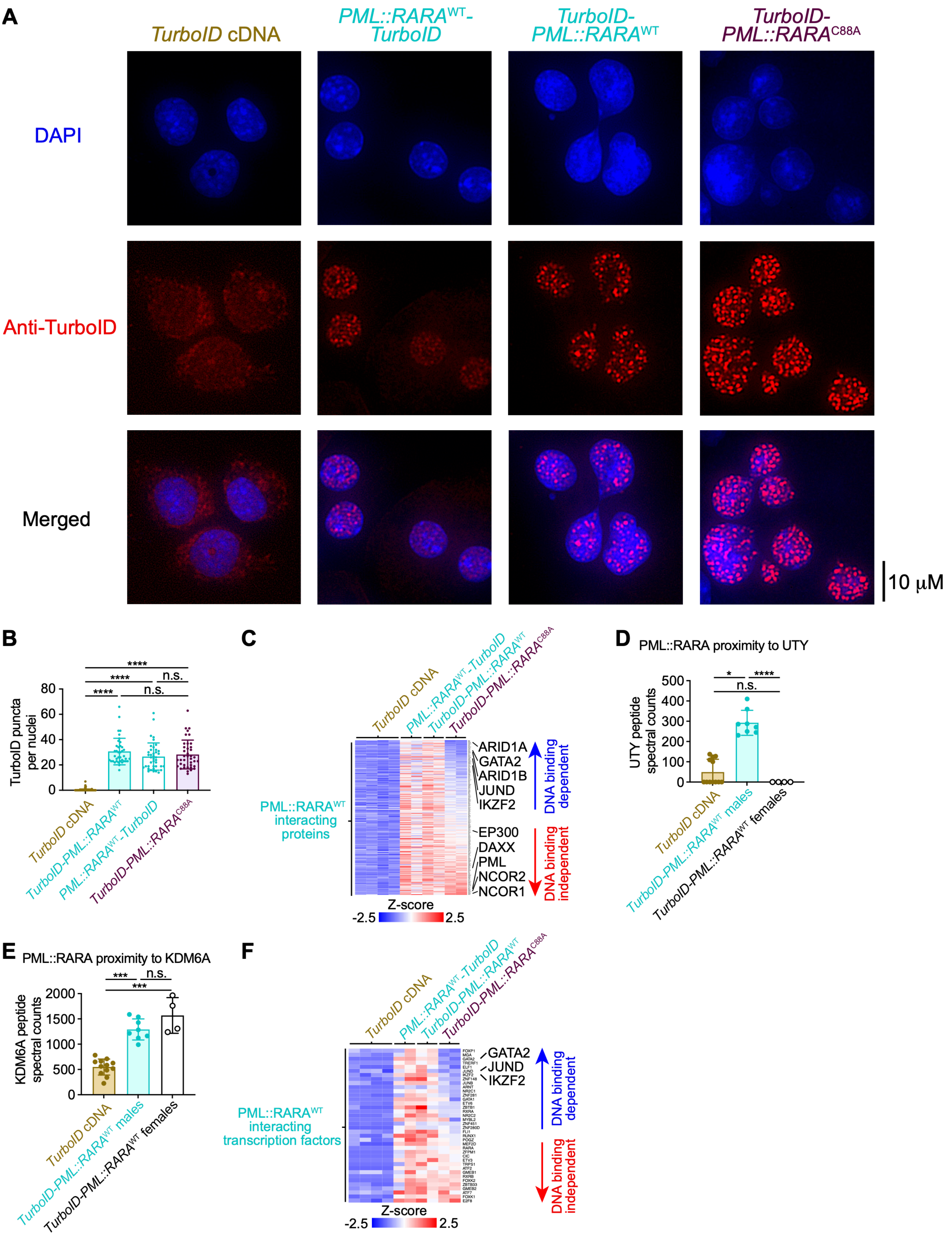
A
TurboID cDNA
PML::RARA^WT-TurboID
TurboID-PML::RARA^WT
TurboID-PML::RARA^C88A
DAPI
Anti-TurboID
Merged
10 μM
B
TurboID puncta per nuclei
TurboID cDNA
TurboID-PML::RARA^WT
PML::RARA^WT-TurboID
TurboID-PML::RARA^C88A
n.s.
C
PML::RARA^WT interacting proteins
ARID1A
GATA2
ARID1B
JUND
IKZF2
EP300
DAXX
PML
NCOR2
NCOR1
DNA binding dependent
DNA binding independent
Z-score
-2.5
2.5
D
PML::RARA proximity to UTY
UTY peptide spectral counts
TurboID-PML::RARA^WT males
TurboID-PML::RARA^WT females
E
PML::RARA proximity to KDM6A
KDM6A peptide spectral counts
F
PML::RARA^WT interacting transcription factors
GATA2
JUND
IKZF2

Fig. S14. PML::RARA-TurboID reorganizes PML nuclear bodies into microspeckles. (A). Immunofluorescence of lineage-depleted mouse bone marrow cells transduced with MSCV-IRES-GFP based retroviruses containing *PML::RARA*^{WT}-TurboID, *TurboID-PML::RARA*^{WT}, *TurboID-PML::RARA*^{C88A}, or *TurboID* cDNA alone. GFP+ cells were flow purified and stained with DAPI (blue) and an anti-TurboID antibody (red). Images are from one sample that is representative of three independent biological replicates. (B) Quantification of the number of PML puncta per nuclei in the cells from panel (A). Data are from three independent biological replicates. ****P < 0.0001, n.s. = not significant by two-way ANOVA. (C) Heat map of the PML::RARA^{WT} interacting proteins ordered by the fold change in interaction with PML::RARA^{WT} (PML::RARA^{WT}-TurboID and TurboID-PML::RARA^{WT}) compared the interaction with TurboID-PML::RARA^{C88A}. Proteins labeled with TurboID alone are passively plotted. Each column represents an independent biological replicate, and are representative of a total of n = 8 for *TurboID* cDNA, n = 4 for PML::RARA^{WT}-TurboID, n = 4 for TurboID-PML::RARA^{WT}, and n = 4 for TurboID-PML::RARA^{C88A} (see Fig. 6 for the remaining replicates). (D-E) Normalized peptide spectral counts of UTY (D) and KDM6A (E) following proximity labeling in cells transduced with *TurboID* cDNA alone or *TurboID-PML::RARA*^{WT}. Donor cells for the transduction with *TurboID-PML::RARA*^{WT} are designated by sex. ****FDR < 1e-60, ***FDR < 1e-10, *FDR $\leq$ 0.05, n.s. = not significant by edgeR (18). (F) Heat map of the transcription factors interacting with PML::RARA^{WT}, ordered by the fold change in interaction with PML::RARA^{WT} (PML::RARA^{WT}-TurboID and TurboID-PML::RARA^{WT}) compared the interaction with TurboID-PML::RARA^{C88A}. *TurboID* cDNA alone is passively plotted.

Terminology	Description	Application
PML::RARA-V5	MSCV - *PML::RARA* with V5 tag on C-terminus	ChIP-seq, CUT&RUN
V5-PML::RARA	MSCV - *PML::RARA* with V5 tag on N-terminus	ChIP-seq
PML::RARA-TurboID	MSCV - *PML::RARA* with TurboID on C-terminus	Protein interactions
TurboID-PML::RARA	MSCV - *PML::RARA* with TurboID on N-terminus	Protein interactions
PML::RARA^{C88A}	MSCV - *PML::RARA* with DNA binding mutation	ChIP-seq, CUT&RUN, ATAC-seq, scRNA-seq
Ctsg-PML::RARA	*PML::RARA* knock-in mouse model	ATAC-seq, RNA-seq
Gata2-V5	MSCV - *Gata2* with V5 tag on C-terminus	ChIP-seq
MSCV	Retrovirus to express *PML::RARA, Gata2*	ChIP-seq, CUT&RUN, ATAC-seq, scRNA-seq, protein interactions
Empty vector	Empty MSCV-based retrovirus	ChIP-seq, CUT&RUN, ATAC-seq, scRNA-seq
Thy1.1	Cell surface marker for MSCV transduction	ChIP-seq, CUT&RUN
TurboID	Highly active biotin ligase	Protein interactions

Table S1. Description and applications of the terminology used in the study. MSCV is the murine stem cell virus used to retrovirally express *PML::RARA* and *Gata2* constructs in primary hematopoietic cells. ChIP-seq = chromatin immunoprecipitation sequencing, ATAC-seq = assay for transposase-accessible chromatin with sequencing, and scRNA-seq = single cell RNA sequencing.

Guide RNA	Guide RNA sequence
Rosa26 intron 1 CRISPR/Cas9 guide RNA 1	TGCAAGTTGAGTCCATCCGC
Rosa26 intron 1 CRISPR/Cas9 guide RNA 2	GGAACACCACCTGACGGGAG
Gata2 upstream CRISPR/Cas9 guide RNA	ATAAGAACGTAATCGCCCAC
Gata2 downstream CRISPR/Cas9 guide RNA	CTGGACACCGATATTGCACT

Primer	Primer Sequence
Gata2 targeted PCR sequencing Forward primer 1	CACTCTTTCCCTACACGACGCTCTTCCGATCTGTTTGAGGTTCAGGGGGCTT
Gata2 targeted PCR sequencing Reverse primer	GTGACTGGAGTTCAGACGTGTGCTCTTCCGATCTGTTACTTCCGGTTAGGGTGCT
Gata2 targeted PCR sequencing Forward primer 2	CACTCTTTCCCTACACGACGCTCTTCCGATCTTGTCCCAAGCTTCGATTCTGT
Rosa26 targeted PCR sequencing Forward primer	CACTCTTTCCCTACACGACGCTCTTCCGATCTAGGGAGGGTCAGCGAAAGTA
Rosa26 targeted PCR sequencing Reverse primer 1	GTGACTGGAGTTCAGACGTGTGCTCTTCCGATCTAGGCATTCATGGGAGTGGAAAG
Rosa26 targeted PCR sequencing Reverse primer 2	GTGACTGGAGTTCAGACGTGTGCTCTTCCGATCTACTCAAGTCGGAAACGTGCT

cDNA	DNA sequence
V5 epitope tag	ATGGGTAAGCCTATCCCTAACCCTCTCCTCGGTCTCGATTCTACG
TurboID	ATGAAAGACAATACTGTGCCTCTGAAGCTGATCGCTCTCCTGGCTAATGGCGAGTTCCAT<br>AGTGGCGAACAGCTGGGAGAAACCCTGGGCATGTCCAGGGCCGCTATCAACAAGCACAT<br>TCAGACTCTGCGCGACTGGGGCGTGGACGTGTTCACCGTGCCCGGAAAGGGCTACTCTC<br>TGCCCGAGCCTATCCCGCTGCTGAACGCTAAACAGATTCTGGGACAGCTGGACGGCGGG<br>AGCGTGGCAGTCCTGCCTGTGGTCGACTCCACCAATCAGTACCTGCTGGATCGAATCGG<br>CGAGCTGAAGAGTGGGGATGCTTGCATTGCAGAATATCAGCAGGCAGGGAGAGGAAGC<br>AGAGGGAGGAAATGGTTCTCTCCTTTTGGAGCTAACCTGTACCTGAGTATGTTTTGGCGC<br>CTGAAGCGGGGACCAGCAGCAATCGGCCTGGGCCCGGTCATCGGAATTGTCATGGCAG<br>AAGCGCTGCGAAAGCTGGGAGCAGACAAGGTGCGAGTCAAATGGCCCAATGACCTGTAT<br>CTGCAGGATAGAAAGCTGGCAGGCATCCTGGTGGAGCTGGCCGGAATAACAGGCGATG<br>CTGCACAGATCGTCATTGGCGCCGGGATTAACGTGGCTATGAGGCGCGTGGAGGAAAG<br>CGTGGTCAATCAGGGCTGGATCACACTGCAGGAAGCAGGGATTAACCTGGACAGGAATA<br>CTCTGGCCGCTACGCTGATCCGAGAGCTGCGGGCAGCCCTGGAACTGTTCGAGCAGGA<br>AGGCCTGGCTCCATATCTGCCACGGTGGGAGAAGCTGGATAACTTCATCAATAGACCCG<br>TGAAGCTGATCATTGGGGACAAAGAGATTTTCGGGATTAGCCGGGGGATTGATAAACAG<br>GGAGCCCTGCTGCTGGAACAGGACGGAGTTATCAAACCCTGGATGGGCGGAGAAATCA<br>GTCTGCGGTCTGCCGAAAAG
TurboID flexible linker	GGTGGAGGTGGTTCTGGCGGAGGCGGCTCTGGAGGAGGAGGAAGTGGATCC
Thy1.1	ATGAACCCAGCCATCAGCGTCGCTCTCCTGCTCTCAGTCTTGCAGGTGTCCCGAGGGCA<br>GAAGGTGACCAGCCTGACAGCCTGCCTGGTGAACCAAAACCTTCGCCTGGACTGCCGCC<br>ATGAGAATAACACCAAGGATAACTCCATCCAGCATGAGTTCAGCCTGACCCGAGAGAAGA<br>GGAAGCACGTGCTCTCAGGCACCCTTGGGATACCCGAGCACACGTACCGCTCCCGCGTC<br>ACCCTCTCCAACCAGCCCTATATCAAGGTCCTTACCCTAGCCAACTTCACCACCAAGGAT<br>GAGGGCGACTACTTTTGTGAGCTTCGCGTCTCGGGCGCGAATCCCATGAGCTCCAATAA<br>AAGTATCAGTGTGTATAGAGACAAGCTGGTCAAGTGTGGCGGCATAAGCCTGCTGGTTCA<br>GAACACATCCTGGATGCTGCTGCTGCTGCTTTCCCTCTCCCTCCTCCAAGCCCTGGACTT<br>CATTTCTCTGTGA

Table S2. A list of CRISPR/Cas9 guide RNA sequences, primers for sequencing and quantifying CRISPR/Cas9 genome editing efficiency, and the cDNA sequences of the V5 epitope tag, TurboID, TurboID flexible linker, and Thy1.1 marker.

Datasets

Dataset S1. A subset of the 2,677 PML::RARA^{WT} genomic binding sites in mouse hematopoietic cells by ChIP-seq and CUT&RUN that have a gene within 1 kb. Annotated is the location of each binding site in the mm10 reference genome and the gene(s) within 1 kb.

Dataset S2. A subset of the PML::RARA^{WT} genomic binding sites in human hematopoietic cells by ChIP-seq that have a gene within 1 kb. The location of each binding site in the hg38 reference genome, and the gene(s) within 1 kb, are annotated.

Dataset S3. A list of the differentially expressed genes between GFP+ mouse hematopoietic cells transduced with *PML::RARA*^{WT} or empty vector MSCV-IRES-GFP retroviruses by scRNA-seq.

Dataset S4. A list of the differentially expressed genes between GFP+ mouse hematopoietic cells transduced with *PML::RARA*^{WT} or *PML::RARA*^{C88A} MSCV-IRES-GFP retroviruses by scRNA-seq.

Dataset S5. A list of the differentially expressed genes between GFP+ human hematopoietic cells transduced with *PML::RARA*^{WT} or empty vector MSCV-IRES-GFP retroviruses by scRNA-seq.

Dataset S6. A list of the differentially expressed genes between GFP+ human hematopoietic cells transduced with *PML::RARA*^{WT} or *PML::RARA*^{C88A} MSCV-IRES-GFP retroviruses by scRNA-seq.

Dataset S7. A list of the differentially expressed genes between flow purified promyelocytes from *Ctsg-PML::RARA* vs. WT mice by RNA-seq, with 3 independent biological replicates per condition.

Dataset S8. A subset of the differentially accessible regions between flow purified promyelocytes from *Ctsg-PML::RARA* vs. WT mice by ATAC-seq that have a gene within 1 kb. Annotated is the location of each differentially accessible region in the mm10 reference genome and the gene(s) within 1 kb, and the relative change in DNA accessibility between *Ctsg-PML::RARA* vs. WT promyelocytes.

Dataset S9. Regions called as accessible by MACS 2.0 software in WT promyelocytes but not in *Ctsg-PML::RARA* promyelocytes by ATAC-seq, and a gene within 1 kb. Annotated is the location of each accessible region in the mm10 reference genome and the gene(s) within 1 kb.

Dataset S10. Regions called as accessible by MACS 2.0 software in *Ctsg-PML::RARA* promyelocytes but not in WT promyelocytes by ATAC-seq, with a gene within 1 kb. The location of each accessible region in the mm10 reference genome, and the gene(s) within 1 kb, are annotated

Dataset S11. A subset of the differentially accessible regions between mouse hematopoietic cells transduced with *PML::RARA*^{WT} vs. an empty vector by ATAC-seq that have a gene within 1 kb. Annotated are the locations of each differentially accessible region in the mm10 reference genome, the gene(s) within 1 kb, and the relative change in DNA accessibility between *PML::RARA*^{WT} vs. empty vector transduced cells.

Dataset S12. A subset of the differentially accessible regions between mouse hematopoietic cells transduced with *PML::RARA*^{WT} vs. *PML::RARA*^{C88A} by ATAC-seq with a gene within 1 kb. Annotated are the locations of each differentially accessible region in the mm10 reference genome, the gene(s) within 1 kb, and the relative change in DNA accessibility between *PML::RARA*^{WT} vs. *PML::RARA*^{C88A} transduced cells.

Dataset S13. A subset of the GATA2 genomic binding sites in mouse hematopoietic cells by ChIP-seq with a gene within 1 kb. Annotated are the locations of each binding site in the mm10 reference genome, and the gene(s) within 1 kb.

Dataset S14. A subset of the differentially accessible regions between mouse hematopoietic cells transduced with *Gata2* vs. an empty vector by ATAC-seq and a gene within 1 kb. Annotated are the locations of each differentially accessible region in the mm10 reference genome, the gene(s) within 1 kb, and the relative change in DNA accessibility between *Gata2* vs. empty vector transduced cells.

Dataset S15. A list of the differentially expressed genes between mouse hematopoietic cells transduced with *Gata2* or an empty vector, determined by scRNA-seq.

Dataset S16. A list of the differentially expressed genes by scRNA-seq between *Ctsg-PML::RARA x Cas9-GFP* mouse hematopoietic cells transfected with CRISPR/Cas9 guide RNAs targeting *Gata2,* or *Rosa26* intron 1.

Dataset S17. A list of the PML::RARA^{WT} interacting proteins by TurboID proximity labeling and mass spectrometry. Normalized spectral counts are listed for each protein. Each column represents an independent replicate.

Dataset S18. A list of the DNA-binding-dependent PML::RARA^{WT} interacting proteins by TurboID proximity labeling and mass spectrometry. Normalized spectral counts are listed for each protein. Each column represents an independent replicate.

SI References

1. C. D. S. Katerndahl *et al.*, Antagonism of B cell enhancer networks by STAT5 drives leukemia and poor patient survival. *Nat Immunol* **18**, 694-704 (2017).
2. J. E. Payton *et al.*, High throughput digital quantification of mRNA abundance in primary human acute myeloid leukemia samples. *J Clin Invest* **119**, 1714-1726 (2009).
3. C. D. S. Katerndahl *et al.*, Tumor suppressor function of Gata2 in acute promyelocytic leukemia. *Blood* **138**, 1148-1161 (2021).
4. P. J. Skene, S. Henikoff, An efficient targeted nuclease strategy for high-resolution mapping of DNA binding sites. *Elife* **6** (2017).
5. L. D. Wartman *et al.*, Expression and function of PML-RARA in the hematopoietic progenitor cells of Ctsg-PML-RARA mice. *PLoS One* **7**, e46529 (2012).
6. A. A. Petti *et al.*, A general approach for detecting expressed mutations in AML cells using single cell RNA-sequencing. *Nat Commun* **10**, 3660 (2019).
7. C. B. Cole *et al.*, PML-RARA requires DNA methyltransferase 3A to initiate acute promyelocytic leukemia. *J Clin Invest* **126**, 85-98 (2016).
8. Y. Zhang *et al.*, Model-based analysis of ChIP-Seq (MACS). *Genome Biol* **9**, R137 (2008).
9. D. Y. Wu, D. Bittencourt, M. R. Stallcup, K. D. Siegmund, Identifying differential transcription factor binding in ChIP-seq. *Front Genet* **6**, 169 (2015).
10. Q. H. Li, J. B. Brown, H. Y. Huang, P. J. Bickel, Measuring Reproducibility of High-Throughput Experiments. *Ann Appl Stat* **5**, 1752-1779 (2011).
11. S. Wang *et al.*, Target analysis by integration of transcriptome and ChIP-seq data with BETA. *Nat Protoc* **8**, 2502-2515 (2013).
12. B. Gel *et al.*, regioneR: an R/Bioconductor package for the association analysis of genomic regions based on permutation tests. *Bioinformatics* **32**, 289-291 (2016).
13. S. Ketkar *et al.*, Remethylation of Dnmt3a (-/-) hematopoietic cells is associated with partial correction of gene dysregulation and reduced myeloid skewing. *Proc Natl Acad Sci U S A* **117**, 3123-3134 (2020).
14. S. Heinz *et al.*, Simple combinations of lineage-determining transcription factors prime cis-regulatory elements required for macrophage and B cell identities. *Mol Cell* **38**, 576-589 (2010).
15. J. A. Blake *et al.*, Mouse Genome Database (MGD): Knowledgebase for mouse-human comparative biology. *Nucleic Acids Res* **49**, D981-D987 (2021).
16. J. Chen, E. E. Bardes, B. J. Aronow, A. G. Jegga, ToppGene Suite for gene list enrichment analysis and candidate gene prioritization. *Nucleic Acids Res* **37**, W305-311 (2009).
17. M. H. Kramer *et al.*, Proteomic and phosphoproteomic landscapes of acute myeloid leukemia. *Blood* **140**, 1533-1548 (2022).
18. M. D. Robinson, D. J. McCarthy, G. K. Smyth, edgeR: a Bioconductor package for differential expression analysis of digital gene expression data. *Bioinformatics* **26**, 139-140 (2010).
19. K. Wang *et al.*, PML/RARalpha targets promoter regions containing PU.1 consensus and RARE half sites in acute promyelocytic leukemia. *Cancer Cell* **17**, 186-197 (2010).
20. J. H. Martens *et al.*, PML-RARalpha/RXR Alters the Epigenetic Landscape in Acute Promyelocytic Leukemia. *Cancer Cell* **17**, 173-185 (2010).
21. Y. Tan *et al.*, A PML/RARalpha direct target atlas redefines transcriptional deregulation in acute promyelocytic leukemia. *Blood* **137**, 1503-1516 (2021).
22. A. A. Lane, T. J. Ley, Neutrophil elastase cleaves PML-RARalpha and is important for the development of acute promyelocytic leukemia in mice. *Cell* **115**, 305-318 (2003).

23. T. J. Ley *et al.*, Genomic and epigenomic landscapes of adult de novo acute myeloid leukemia. *N Engl J Med* **368**, 2059-2074 (2013).
24. K. S. Pollard, M. J. Hubisz, K. R. Rosenbloom, A. Siepel, Detection of nonneutral substitution rates on mammalian phylogenies. *Genome Res* **20**, 110-121 (2010).
25. K. D. Johnson *et al.*, Cis-regulatory mechanisms governing stem and progenitor cell transitions. *Sci Adv* **1**, e1500503 (2015).
26. S. Groschel *et al.*, A single oncogenic enhancer rearrangement causes concomitant EVI1 and GATA2 deregulation in leukemia. *Cell* **157**, 369-381 (2014).
